# Construction and validation of a nomogram based on N6‐Methylandenosine‐related lncRNAs for predicting the prognosis of non‐small cell lung cancer patients

**DOI:** 10.1002/cam4.4961

**Published:** 2022-06-21

**Authors:** Wenjing Xiao, Wei Geng, Juanjuan Xu, Qi Huang, Jinshuo Fan, Qi Tan, Zhengrong Yin, Yumei Li, Guanghai Yang, Yang Jin

**Affiliations:** ^1^ Department of Respiratory and Critical Care Medicine, NHC Key Laboratory of Pulmonary Diseases, Union Hospital, Tongji Medical College Huazhong University of Science and Technology Wuhan Hubei China; ^2^ Department of Thoracic Surgery, Union Hospital, Tongji Medical College Huazhong University of Science and Technology Wuhan Hubei China

**Keywords:** long non‐coding RNAs, N6‐methyladenosine, non‐small cell lung cancer, prognostic signature

## Abstract

**Background:**

The N6‐methyladenosine (m^6^A) can modify long non‐coding RNAs (lncRNAs), thereby influencing a wide array of biological functions. However, the prognosis of m^6^A‐related lncRNAs (m^6^ARLncRNAs) in non‐small cell lung cancer (NSCLC) remains largely unknown.

**Methods:**

Pearson correlation analysis was used to identify m^6^ARLncRNAs in 1835 NSCLC patients and with the condition (|Pearson R| > 0.4 and *p* < 0.001). Univariant Cox regression analysis was conducted to explore the prognostic m^6^ARLncRNAs. We filtered prognostic m^6^ARLncRNAs by LASSO regression and multivariate Cox proportional hazard regression to construct and validate an m^6^ARLncRNAs signature (m^6^ARLncSig). We analyzed the correlation between the m^6^ARLncSig score and clinical features, immune microenvironment, tumor mutation burden, and therapeutic sensitivity and conducted independence and clinical stratification analysis. Finally, we established and validated a nomogram for prognosis prediction in NSCLC patients.

**Results:**

Forty‐one m^6^ARLncRNAs were identified as prognostic lncRNAs, and 12 m^6^ARLncRNAs were selected to construct m^6^ARLncSig in the TCGA training dataset. The m^6^ARLncSig was further validated in the testing dataset, GSE31210, GSE37745, GSE30219, and our NSCLC samples. In terms of m^6^ARLncSig, NSCLC patients were divided into high‐ and low‐risk groups, with significantly different overall survival (OS), clinical features (age, sex, and tumor stage), tumor‐infiltrating immune cells, chemotherapeutic sensitivity, radiotherapeutic response, and biological pathways. Moreover, m^6^ARLncSig independently predicted the OS of NSCLC patients. Finally, the robustness and clinical practicability for predicting NSCLC patient prognosis was improved by constructing a nomogram containing the m^6^ARLncSig, age, gender, and tumor stage.

**Conclusions:**

Our study demonstrated that m^6^ARLncSig could act as a potential biomarker for evaluating the prognosis and therapeutic efficacy in NSCLC patients.

## Graphical abstract

The study flow chart and correlation of m^6^ARLncRNAs and m^6^A regulators.

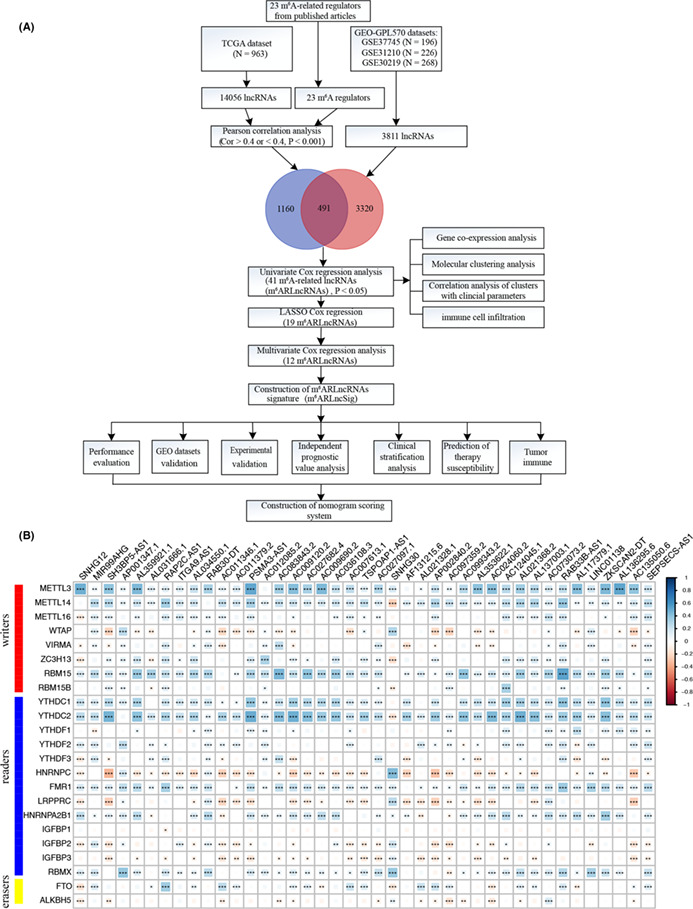

## 1 INTRODUCTION

Lung cancer represents the leading cause of cancer‐related deaths worldwide, with non‐small cell lung cancer (NSCLC) accounting for 85% of lung cancer.^1^, ^2^ Owing to late‐stage disease presentation, high metastasis, tumor relapse, and resistance to therapies, the 5‐year survival rate stands somewhere remains poor at 10%–20%.^3^, ^4^ Some studies have shown that clinical indicators are intimately associated with the prognosis of patients, such as age, smoking status, and stage classification.^5^ However, the prognosis among NSCLC patients with the same stage varies greatly, which boils down to high heterogeneity and differential biological characteristics of individual tumors. Hence, accurately judging the prognosis of patients based on molecular prognostic markers and identifying patients who are at high‐risk is of importance.

The N6‐methyladenosine (m^6^A), that is, the methylation at the 6th N atom of adenine, is the most common modification of mRNAs and non‐coding RNAs (ncRNAs) and widely seen in eukaryotic species.^6^, ^7^, ^8^ As an invertible and dynamic RNA modification, the m^6^A‐regulated process is determined by three kinds of regulators including “writers” (methyltransferases), “readers” (signal transducers), and “erasers” (demethylases). The m^6^A methylation modification is closely linked with the regulation of splicing, export, and stabilization of RNA.^7^, ^9^ It has been reported that the aberrant expression of m6A regulators is implicated in the development and progression of various malignancies.^10^, ^11^, ^12^ For example, ALKBH5 promoted tumor cell proliferation by destabilizing IGF2BPs target genes and worsening the prognosis of NSCLC patients.^13^

Studies have demonstrated that m^6^A methylation can extensively modify long non‐coding RNAs (lncRNAs), a class of transcripts over 200 nucleotides long that possess no or only limited protein‐coding potential.^8^, ^14^ It has been reported that lncRNAs can regulate gene expression and play vital roles in cellular proliferation, migration, and survival.^12^ As a result, m^6^A modification affected the expression levels and functions of lncRNAs, consequently controlling and influencing cancer progression. For example, upregulated LINC00958 modified by METTL3 facilitates HCC cell migration and invasion by sponging miR‐3619‐5p.^15^ Therefore, probing m^6^A modification of lncRNA in NSCLC may help find the new molecular mechanism and identify a prognostic biomarker for clinical application. Up to now, m^6^A‐related lncRNAs (m^6^ARLncRNAs) signatures have been constructed to predict the prognosis of lung adenocarcinoma (LUAD) and lung squamous cell carcinoma (LUSC).^16^, ^17^ However, the role of m^6^A modification regulators in the regulation of lncRNAs in NSCLC has not been fully elucidated.

In the present study, we examined the prognostic relevance of m^6^ARLncRNAs in 1835 NSCLC patients and constructed an m^6^ARLncRNAs prognostic signature (m^6^ARLncSig) for predicting the overall survival (OS) of NSCLC patients.

## 2 MATERIALS AND METHODS

### 2.1 Research roadmap

**FIGURE 1:**
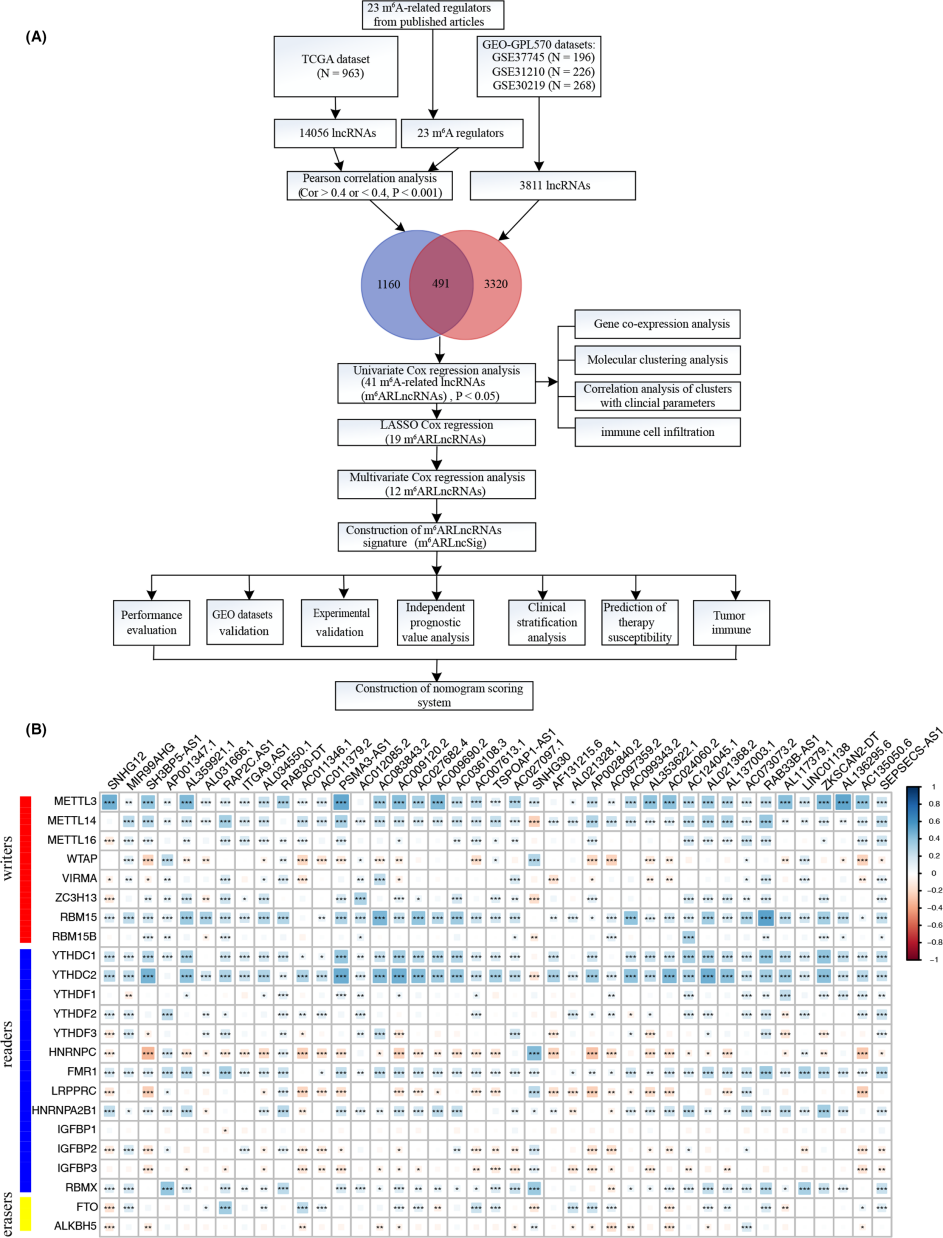
(A) The study flow chart. (B) The correlation of 41 prognostic m^6^ARLncRNAs and 23 m^6^A regulators with the |Pearson R| > 0.4 and *p* < 0.001. *p < 0.05, **p < 0.01, and ***p < 0.001.

The research procedure of this study is depicted in Figure 1A. After data processing, co‐expression analyses between lncRNAs and m^6^A regulators were utilized to identify m^6^A‐related lncRNAs (m^6^ARLncRNAs) with the conditions (|Pearson R| > 0.4 and *p* < 0.001). Univariate Cox regression analyses were employed to screen out prognostic m^6^ARLncRNAs. The molecular clustering patterns were explored based on the expression levels of prognostic m^6^ARLncRNAs. Then, the NSCLC patients were divided, 1:1 at random, into two datasets: a training set and a testing set. We constructed a multivariable Cox regression model following variables selection using LASSO regression, which generated a prognostic m^6^A‐related lncRNAs signature (m^6^ARLncSig) for predicting the overall survival of NSCLC patients. Subsequently, m^6^ARLncSig was subjected to an assessment, covering the receiver operating characteristic (ROC) curve, external datasets validation, experimental validation with NSCLC samples, independent prognostic value analyses, and clinical stratification analyses. Finally, a nomogram scoring system was established to improve the practicability of the m^6^ARLncSig as a prognostic predictor for NSCLC patients.

### 2.2 Data processing

The whole workflow of this study is presented in Figure 1A. In the study, a total of 23 recognized m^6^A regulators were obtained from the previous publications, including writers (METTL3, METTL14, METTL16, WTAP, VIRMA, ZC3H13, RBM15, RBM15B), erasers (FTO and ALKBH5), and readers (YTHDC1, YTHDC2, YTHDF1, YTHDF2, YTHDF3, HNRNPC, FMR1, LRPPRC, HNRNPA2B1, IGFBP1, IGFBP2, IGFBP3, and RBMX). These mRNA and lncRNA transcriptional profiles of lncRNAs and m^6^A regulators and the clinical features of 1145 patients were downloaded and extracted from The Cancer Genome Atlas (TCGA) database (https://portal.gdc.cancer.gov/). GTF files were downloaded from Ensembl (http://asia.ensembl.org/index.html) for differentiating mRNAs and lncRNAs. And mRNAs and lncRNAs symbols were annotated using the HUGO Gene Nomenclature Committee (HGNC2) database (https://www.genenames.org/). Some clinical samples were excluded from the criteria due to the absence of survival data or a short survival time (<30‐day). Lastly, 963 patients were included in the study, equally randomized into the training dataset for m^6^ARLncSig model construction and the testing dataset for model validation. The baseline clinical data of NSCLC patients are listed in Table 1. For external validation, detailed clinical and survival data of three independent datasets, including GSE37745 (*N* = 196), GSE30219 (*N* = 268), and GSE31210 (*N* = 226) with large samples from the GPL570 Affymetrix, were downloaded from Gene Expression Omnibus (GEO) (https://www.ncbi.nlm.nih.gov/geo/query/acc.cgi). The Affymetrix HG‐U133_Plus 2.0 platform probes were re‐annotated to gene symbols by matching the sequence files (HG‐U133_Plus_2 Probe Sequences, FASTA format, August 20, 2008) of the probe sets and the annotation files of GENCODE (release 37).

**TABLE 1:** Clinical information of patients with NSCLC in the TCGA cohort

Covariates	Type	Total (*N* = 963)	Training dataset (*N* = 483)	Testing dataset (*N* = 480)	*p‐*value
Age (%)	≤65	414 (42.99%)	207 (42.86%)	207 (43.12%)	1
	>65	534 (55.45%)	267 (55.28%)	267 (55.62%)	
	Unknow	15 (1.56%)	9 (1.86%)	6 (1.25%)	
Gender (%)	Female	384 (39.88%)	194 (40.17%)	190 (39.58%)	0.9055
	Male	579 (60.12%)	289 (59.83%)	290 (60.42%)	
Tumor stage (%)	Stage I–II	760 (78.92%)	391 (80.95%)	369 (76.88%)	0.2124
	Stage III–IV	191 (19.83%)	88 (18.22%)	103 (21.46%)	
	Unknow	12 (1.25%)	4 (0.83%)	8 (1.67%)	
T stage (%)	T1–2	810 (84.11%)	398 (82.4%)	412 (85.83%)	0.2479
	T3–4	150 (15.58%)	82 (16.98%)	68 (14.17%)	
	Unknow	3 (0.31%)	3 (0.62%)	0 (0%)	
M stage (%)	M0	717 (74.45%)	347 (71.84%)	370 (77.08%)	0.7209
	M1	30 (3.12%)	13 (2.69%)	17 (3.54%)	
	Unknow	216 (22.43%)	93 (19.38%)	123 (25.47%)	
N stage (%)	N0	617 (64.07%)	313 (64.8%)	304 (63.33%)	0.7131
	N1–3	331 (34.37%)	163 (33.75%)	168 (35%)	
	Unknow	15 (1.56%)	7 (1.45%)	8 (1.67%)	

*Note*: Chi‐squared test, *p* < 0.05 means significantly different.

### 2.3 Identification of m^6^A‐related lncRNAs

Pearson correlation analysis was performed to identify the potential m^6^ARLncRNAs with the conditions (|Pearson R| > 0.4 and *p* < 0.001) based on the expression of lncRNA and m^6^A regulators in the TCGA dataset using “limma” and “corrplot” packages. Then, we filtered the m^6^ARLncRNAs by taking the intersection of lncRNAs between the TCGA and GEO datasets.

### 2.4 Univariate Cox regression analysis

Univariate Cox regression analysis was conducted to screen the differential m^6^ARLncRNAs based on the expression levels of m^6^ARLncRNAs and OS of NSCLC patients in the TCGA dataset (*p* < 0.05) using the “survival” package.

### 2.5 Gene co‐expression analysis

Co‐expression analyses were conducted to visualize the correlation of lncRNAs obtained from univariate Cox regression analysis with 23 m^6^A regulators using “corrplot”, “psych”, “ggthemes”, and “Hmisc” packages.

### 2.6 Consensus unsupervised clustering analysis

We employed consensus unsupervised clustering analysis to explore distinct molecular patterns based on the expression of m^6^ARLncRNAs obtained from univariate Cox regression analysis using “ConsensusClusterPlus” and “ggplot2” packages.^18^

### 2.7 Relationship of consensus clustering and the clinical features, outcomes, and immune infiltration levels in NSCLC patients

We first compared the distribution of age, gender, survival status, and stage among different clusters and presented the results utilizing R software's “pheatmap” package. Subsequently, we calculated the OS of NSCLC patients among different clusters by employing the Kaplan–Meier method and log‐rank test with the “survival” and “survminer” packages. Then, we used CIBERSORT (https://cibersort.stanford.edu/) to determine the change in immune infiltrating levels quantitatively.^19^

### 2.8 Gene set enrichment analysis (GSEA)

We conducted a GSEA functional enrichment analysis of their mRNA partners among high‐ and low‐risk groups using GSEA software from the Broad Institute (http://software.broadinstitute.org/gsea/index.jsp).^20^ A false discovery rate (FDR) of <0.25 and a *p* < 0.05 were considered statistically significant.

### 2.9 Construction of prognostic m^6^ARlncRNA signature (m^6^ARLncSig)

The least absolute shrinkage and selection operator (LASSO) Cox regression algorithm was conducted to shrink differential m^6^ARLncRNAs obtained from univariate Cox regression analysis with “caret”, “glmnet”, and “survival” packages. Then the screened m^6^ARLncRNAs were further subjected to multivariate Cox proportional hazard regression analysis to obtain the optimal candidates, and the prognostic m^6^ARLncSig model was constructed in the TCGA training dataset. The receiver operating characteristic (ROC) analysis was conducted and the areas under curve (AUC) value was calculated to evaluate the performance of the m^6^ARLncSig using the “survivalROC” package. The formula calculated the m6ARLncSig score for the signature:

$$m^{6}\text{ARLncSig}=\sum_{i=1}^{n}\text{coef}\times \mathrm{Xi}.$$

Where “coef” was the regression coefficient and “Xi” was the expression levels of m^6^ARLncRNAs. In terms of the median m^6^ARLncSig score as the cut‐off point, NSCLC patients were divided into high‐ and low‐risk groups. The OS of NSCLC patients between two groups was estimated using the Kaplan–Meier method with log‐rank test using “survival” and “survminer” packages.

### 2.10 External validation and model comparison

Three GEO datasets, including GSE37745, GSE30219, and GSE31210, were used to validate the prognostic performance of the m6ARLncSig further. The m^6^ARLncSig score of NSCLC patients was calculated based on the above formula. Survival curves of the two groups were generated by “survival” and “survminer” packages. For model comparison, we retrieved four prognostic signatures of LUAD or LUSC constructed by similar methods previously and calculated their risk score according to their formula, respectively.^16^, ^17^, ^21^, ^22^ Then, we drew the ROC curves and calculated AUCs to compare the prediction performance of our models with the previous models.

### 2.11 Experimental validation with NSCLC samples by quantitative real‐time polymerase chain reaction (qRT‐PCR)

The ethical committee approved the human tissue investigation of Tongji Medical College, Huazhong University of Science and Technology (protocol: 2010‐S202), which was performed in compliance with the Declaration of Helsinki's standards, and all patients provided informed consent. We included 46 paired tumor tissues and adjacent normal tissues from pathologically and clinically confirmed NSCLC patients who had undergone surgical treatments at the Department of Thoracic Surgery of Wuhan Union Hospital from 2014 to 2019. The exclusion criteria were applied: (i) Age < 18 years and age > 80 years; (ii) without complete follow‐up information; (iii) survival time (< 30 days); (iv) patients who received preoperative radiotherapy and chemotherapy before surgery. The clinicopathological features and follow‐up information of these tissue samples are presented in Table S1.

Total RNA from samples was extracted by using Trizol reagent (Invitrogen, Carlsbad, CA, United States) according to the instructions and cDNA was synthesized using a reverse transcription kit (Takara, Dalian, China). Quantification of m^6^ARlncRNAs was conducted using an SYBR Green PCR Kit (Takara) and Real‐Time PCR System (Applied Biosystems, Carlsbad, CA, USA). The corresponding primers of 12 m^6^ARlncRNAs were listed in Table S2.

### 2.12 Independence and clinical stratification analysis of m^6^ARLncSig

To know whether m^6^ARLncSig could serve as a prognostic factor independent of other clinical factors, including age, gender, and tumor stage, univariate and multivariate Cox regression analyses were performed in the training dataset, testing dataset, GSE31210, GSE30219, and GSE37745 using the “survival” package, respectively. Clinical stratification analysis was performed to validate the prognostic performance of m6ARLncSig further. NSCLC patients in the TCGA dataset were stratified into a young‐patient group aged ≤ 65 (*N* = 414) and an old‐patient group aged >65 (*N* = 534). NSCLC patients were divided into a male‐patient group (*N* = 579) and a female‐patient group (*N* = 384). NSCLC patients were also stratified into an early‐stage group (tumor stage I–II, *N* = 760) and a late‐stage group (tumor Stage III–IV, *N* = 191). Kaplan–Meier method and log‐rank test were used to estimate the OS between different subgroups with the “survival” and “survminer” packages.

### 2.13 Significance of the m^6^ARLncSig in the clinical treatment

To estimate the role of m^6^ARLncSig in NSCLC treatment efficacy prediction, the IC50 of common chemotherapy and molecular targeted drugs therapeutic effects administered in NSCLC patients, such as etoposide, paclitaxel, lenalidomide, docetaxel, methotrexate, erlotinib, and gefitinib between high‐ and low‐risk groups were assessed and drawn with “pRRophetic” and “ggpubr” packages.^23^

### 2.14 Tumor mutation burden (TMB) analysis

The somatic mutation data called simple nucleotide variation (VarScan version) as raw mutation count of NSCLC were downloaded from the TCGA database. The TMB level was evaluated according to the following formula: TMB = Total mutation count /the exome size. The total mutation count was the sum of mutation counts, including missense mutation, nonsense mutation, frame shift deletes, frame shift inserts, in frame deletes, etc. We used 38 Mb as the estimate of the exome size. Then, the TMB level between high‐ and low‐risk groups was analyzed using the Mann–Whitney *U* test and visualized by boxplot with “ggpubr” package.

### 2.15 Establishment and validation of a nomogram scoring system

A nomogram was constructed internally in the training dataset and externally validated in the testing dataset and the whole TCGA dataset for predicting 3‐ and 5‐year survival in terms of multivariate Cox regression analysis to evaluate the independent prognostic significance of m^6^ARLncSig and clinical variables. The ROC curve and calibration plot were drawn. The concordance index (C‐index) was calculated to estimate the nomogram scoring system's predictive accuracy and discriminative capability using the “survivalROC” package.

### 2.16 Statistical analysis

The Chi‐squared test and Fisher's exact test were used to assess the differences in categorical data between different datasets and groups. The Mann–Whitney *U* test or Student *t*‐test to compare the quantitative data. A *p* < 0.05 was considered to be statistically significant. The statistical analyses were performed using R version 4.0.2 (https://www.r‐project.org) with the corresponding functional packages or Graphpad Prism 8 (GraphPad Software, San Diego, USA).

## 3 RESULTS

### 3.1 Identification of m^6^ARLncRNAs in NSCLC patients

Figure 1A shows the flow chart of the study. We identified 14,056 and 3811 lncRNAs derived from the TCGA and GEO datasets. Then, we conducted the Pearson correlation analysis based on the expression levels of lncRNAs and m^6^A regulators to identify the potential m^6^ARLncRNAs with the |Pearson R| > 0.4 and *p* < 0.001 in the TCGA dataset. Consequently, we identified 1651 m^6^ARLncRNAs. Then, 491 m^6^ARLncRNAs were obtained by taking the intersection of GEO and TCGA datasets. Afterward, 41 prognostic m^6^ARLncRNAs were found to bear a significant association with the OS of NSCLC patients, as indicated by univariate Cox regression (Figure S1A). The correlations between 41 m^6^ARLncRNAs and 23 m^6^A regulators in the TCGA dataset were illustrated in Figure 1B and Table S3. The differential expression of 41 m^6^ARLncRNAs in NSCLC tissues relative to normal tissues were depicted in Figure S1B.

### 3.2 Analysis of unsupervised consensus clustering

To understand the effect of m^6^ARLncRNAs on the development of NSCLC, we conducted a consensus unsupervised clustering analysis in the TCGA dataset based on the expression levels of 41 m^6^ARLncRNAs. The plot showed the relative change in area under the cumulative distribution function (CDF) curve in the k‐means (2 to 9) unsupervised clustering of NSCLC (Figure S2A–B). We also showed the tracking plots of subgroups for *k* = 2–9 (Figure S2C). Lastly, two m^6^ARLncRNAs‐associated clusters were determined and dubbed Cluster 1 (*N* = 705) and Cluster 2 (*N* = 258), respectively (Figure S2D). We found that Cluster 2 was significantly correlated with the female, lower N stage, and younger age than Cluster 1 (Figure S3A). And patients in cluster 2 had a longer OS than their counterparts in Cluster 1 (Figure S3B). We also investigated the levels of immune cell infiltration between two clusters. The results showed that cluster 1 had higher infiltration levels of neutrophils, macrophages, and activated memory CD4 T cells (Figure S3C and Figure S4A–C). Conversely, the infiltration levels of monocytes, regulatory T cells, naive B cells, and activated NK cells were higher in Cluster 2 (Figure S4D–G).

### 3.3 Construction of a prognostic signature

**FIGURE 2:**
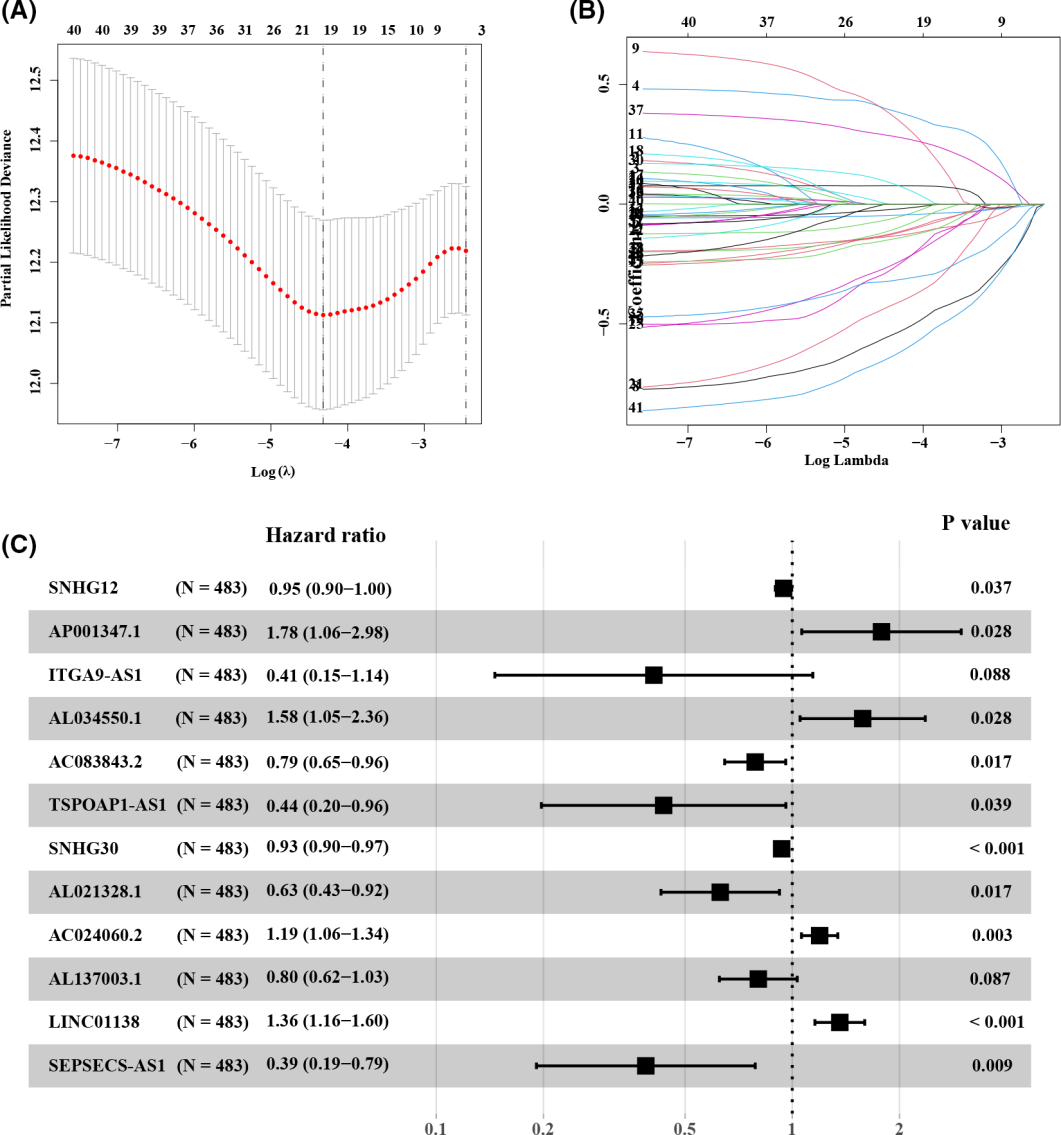
Construction of a prognostic signature. (A) The distribution plot of the partial likelihood deviation of the LASSO coefficient. Twelve variables were retained when the partial likelihood deviation reached the minimum (Log Lambda = −4.3). (B) The distribution plot of the LASSO coefficient. (C) The risk signature's forest plot consisted of 12 m^6^ARLncRNAs based on a multivariate Cox hazard analysis.

To further explore the prognostic implication of m^6^ARLncRNAs in NSCLC, 963 patients were randomly assigned to the training dataset (*N* = 483) and the testing dataset (*N* = 480) at a 1:1 ratio. LASSO Cox regression analysis and multivariate Cox proportional hazard regression for 41 prognostic m^6^ARLncRNAs were conducted to construct further a robust and effective model for prognosis prediction of NSCLC patients (Figure 2A–B). Consequently, 12 m^6^ARLncRNAs were included in the prognostic m^6^ARLncRNAs signature (m^6^ARLncSig) (Figure 2C and Table S4). Among them, AC024060.2, LINC01138, AL034550.1, and AP001347.1 were identified to be high‐risk factors and upregulation of these was indicative of a poor prognosis for NSCLC patients. Contrary, the other m^6^ARLncRNAs, including SNHG12, ITGA9‐AS1, AC083843.2, TSPOAP1‐AS1, SNHG30, AL021328.1, AL137003.1, and SEPSECS‐AS1 were found to be protective factors, indicating a better survival relevance of their upregulated expression.

**FIGURE 3:**
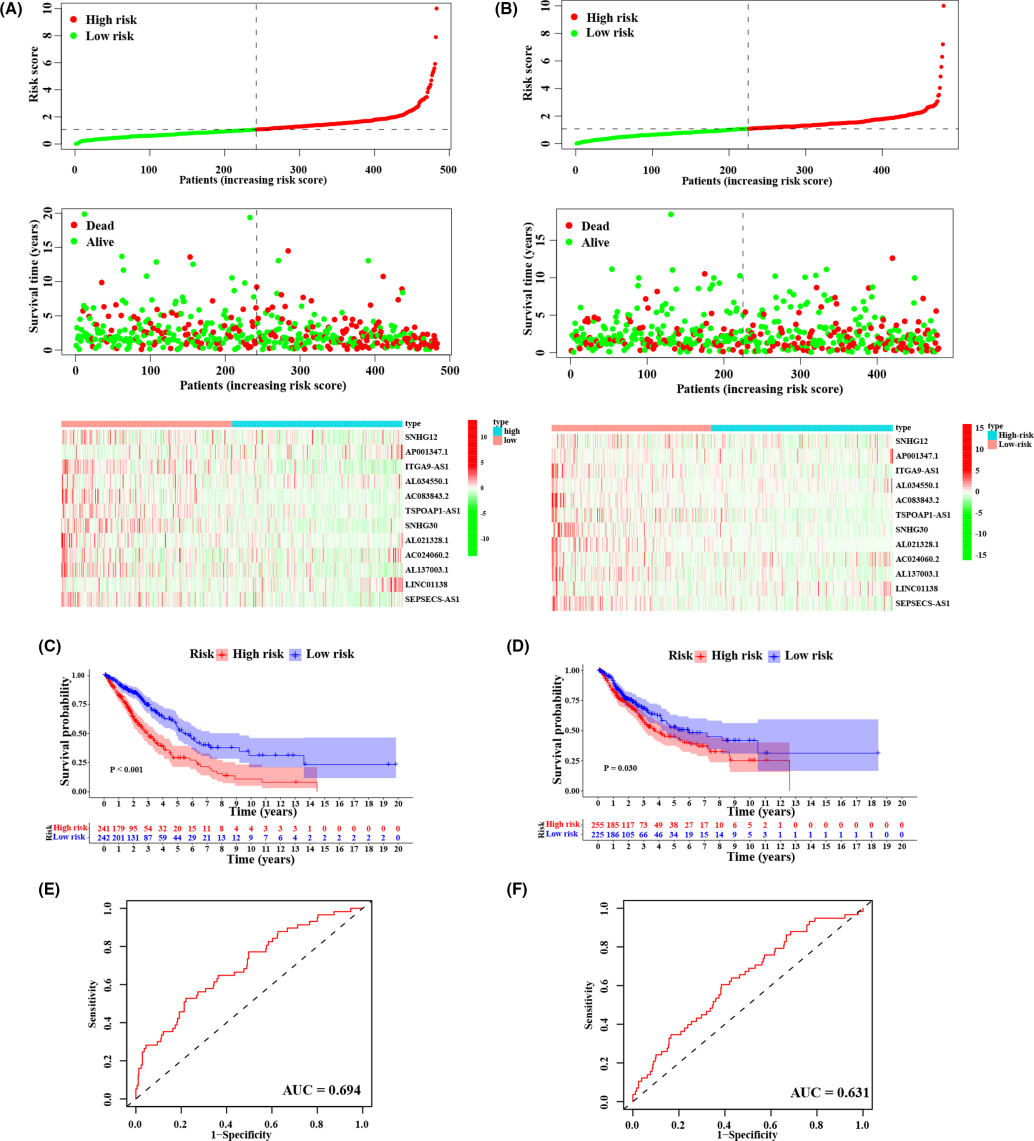
Evaluation and validation of m^6^ARLncRNAs signature (m^6^ARLncSig). (A–B) The distribution of m^6^ARLncSig score, OS, OS status, and heatmap of prognostic m^6^ARLncRNAs in the training dataset (A) and the testing dataset (B). (C–D) Kaplan–Meier survival curves of NSCLC patients in the high‐ and low‐risk groups separated by m^6^ARLncSig score in the training dataset (C) and the testing dataset (D), respectively. (E–F) ROC curves of the prognostic model for predicting the 1‐year survival in the training dataset (E) and the testing dataset (F), respectively.

Subsequently, with the median m^6^ARLncSig score as the cut‐off, patients in the training and testing dataset were divided into high‐ and low‐risk groups, respectively. The distribution of the m^6^ARLncSig scores, OS, OS status, and expression profiles of m^6^ARLncRNAs in the training and the testing datasets was given in Figure 3A–B. Kaplan–Meier survival analysis revealed that patients in the low‐risk group had more favorable outcomes than those in the high‐risk group and the AUC of the ROC curves was 0.694 and 0.631 for the 1‐year OS prediction, whose median score was 1.070 and 1.125 in the training dataset and testing dataset, respectively (Figure 3C–F). Collectively, our results suggested that m^6^ARLncSig promised to be a good survival predictor.

### 3.4 External and experimental validation and predictability comparison

**FIGURE 4:**
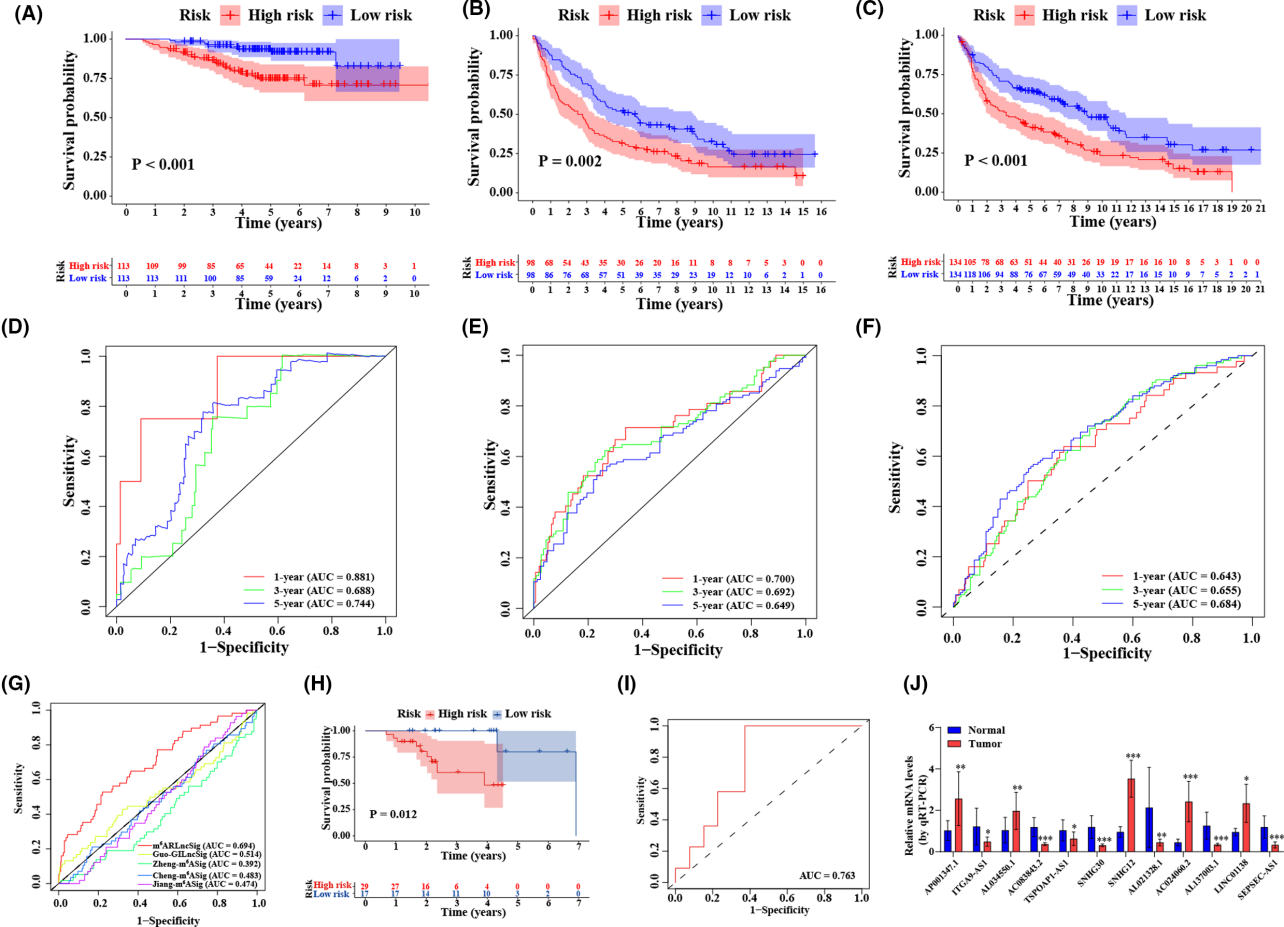
External and experimental validation and model comparison. (A–C) Kaplan–Meier survival curves of patients in the high‐ and low‐risk groups separated by m^6^ARLncSig in GSE31210 (A), GSE37745 (B), and GSE30219 (C), respectively. (D–F) ROC curves of the prognostic model for predicting the 1‐, 3‐ and 5‐year survival in the GSE31210 (D), GSE37745 (E), and GSE30219 (F). (G) ROC curves for 1‐year survival prediction of the m^6^ARLncSig and the other existing signatures, respectively. (H) Kaplan–Meier survival curve in the high‐ and low‐risk groups identified by the determined cut‐off point in the fresh 46 NSCLC tissues. (I) ROC curves of m^6^ARLncSig for 3‐year survival prediction of patients in the NSCLC tissues. (J) qRT‐PCR assay revealing the expression levels of 12 m^6^ARLncRNAs in tumor tissues compared with those in the adjacent normal tissues. *p < 0.05, **p < 0.01, and ***p < 0.001.

To further determine the prognostic significance of the m^6^ARLncSig, we retrieved three GEO datasets, including GSE31210, GSE37745, and GSE30219, from the GPL570 microarray platform. According to the formula mentioned above, the Kaplan–Meier survival analysis indicated that all the patients in the low‐risk group had a significantly longer survival time compared with the high‐risk group in GSE31210 (*p* < 0.001), GSE37745 (*p* = 0.002), and GSE30219 (*p* < 0.001), respectively (Figure 4A–C). The ROC analysis revealed an acceptable prognostic value for NSCLC patients (in the GSE31210 dataset: 1‐year AUC = 0.881, 3‐year AUC = 0.688, 5‐year AUC = 0.744; in the GSE37745 dataset: 1‐year AUC = 0.700, 3‐year AUC = 0.692, 5‐year AUC = 0.649; in the GSE30219 dataset: 1‐year AUC = 0.643, 3‐year AUC = 0.655, 5‐year AUC = 0.684) (Figure 4D–F). Moreover, we conducted a predictability comparison between our m^6^ARLncSig and recently reported prognostic lncRNA signatures. The results showed that our m^6^ARLncSig with an AUC of ROC for the 1‐year OS of 0.694 outperformed better than Jiang‐m^6^ASig (AUC = 0.474) and Guo‐GILncSig (AUC = 0.514), Zheng‐m^6^ASig (AUC = 0.392) and Cheng‐m^6^ASig (AUC = 0.483) in predicting patients' survival (Figure 4G). Additionally, the Kaplan–Meier analysis identified by the cut‐off point also showed that patients in the low‐risk group had better survival outcomes than patients in the high‐risk group in 46 NSCLC tissues using qRT‐PCR, with the AUC was 0.763 for the three‐year survival prediction of patients (*p* = 0.012, Figure 4H–I). Furthermore, we detected the expression levels of 12 m^6^ARLncRNAs in 13 pairs of NSCLC samples. The expression levels of AC024060.2, LINC01138, AL034550.1, SNHG12, and AP001347.1 were upregulated in tumor tissues compared with those in the adjacent normal tissues (Figure 4J). The remaining m^6^ARLncRNAs exhibited downregulation in tumor tissues.

### 3.5 Correlation between m^6^ARLncSig **and clinical** features

**FIGURE 5:**
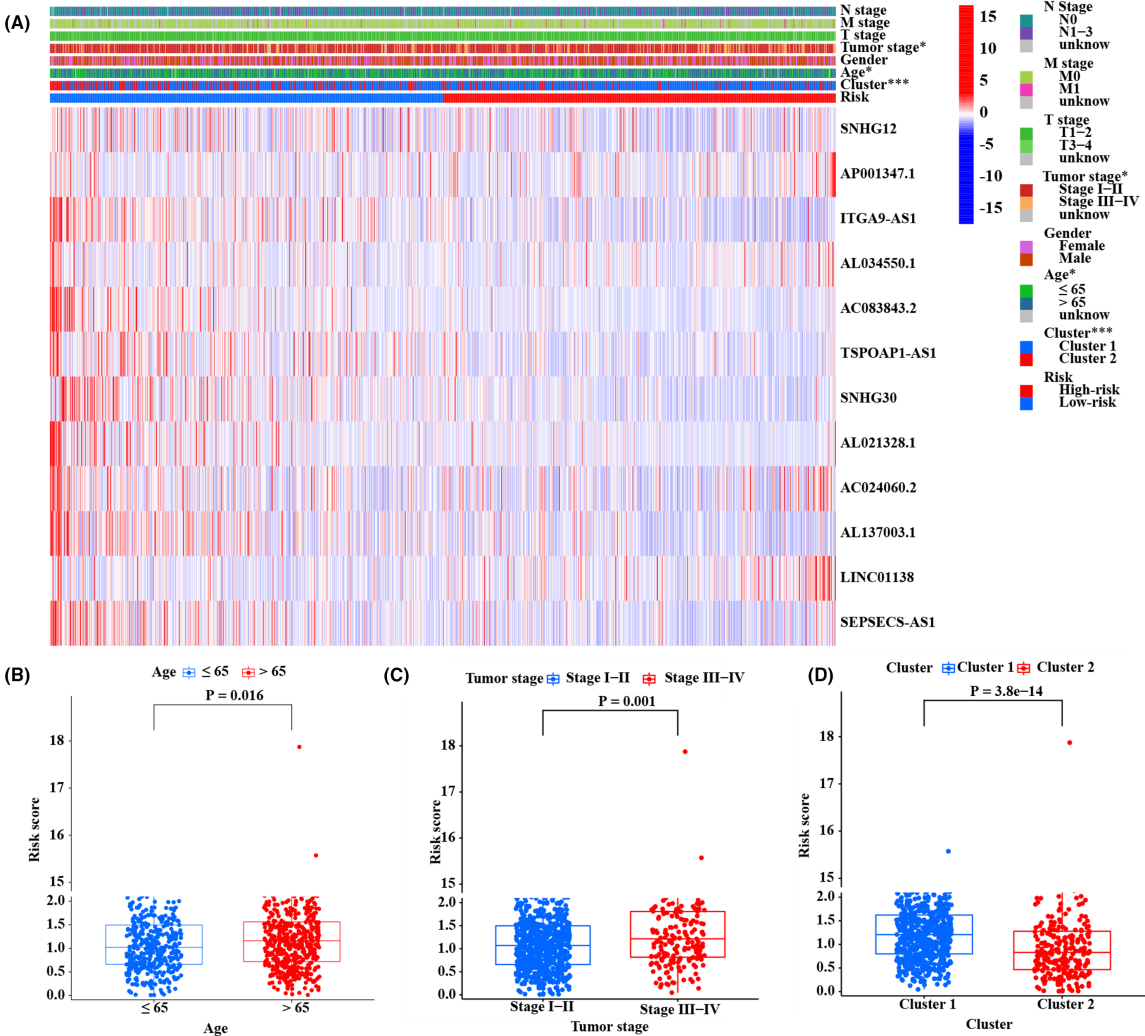
The correlation analysis between m^6^ARLncSig score and clinical features. (A) The heatmap showing the correlation between m^6^ARLncRNAs and clinical characteristics. (B–D) The distribution of m^6^ARLncSig score in NSCLC patients aged ≤65 or >65 (B), patients with tumor Stage I–II or III–IV (C), and patients in Cluster 1 or Cluster 2 (D). *p < 0.05, **p < 0.01, and ***p < 0.001.

Then, we tried to find the correlation between m^6^ARLncSig and clinical features. Our results showed that m^6^ARLncSig was significantly correlated with age, tumor stage, and molecular clustering patterns (*p* < 0.05, Figure 5A). Briefly, patients in the advanced‐age group tended to have significantly higher m^6^ARLncSig scores than those in the young‐patient group (*p* = 0.016, Figure 5B). Patients at the advanced tumor stage had significantly higher m^6^ARLncSig scores than those at the early stage (*p* = 0.001, Figure 5C). Moreover, patients in Cluster 1 had significantly higher m^6^ARLncSig scores than patients in Cluster 2 (*p* < 0.001, Figure 5D).

### 3.6 Gene set enrichment analysis (GSEA)

To identify the underlying biological pathways of m^6^ARlncRNAs, GSEA was employed to conduct between high‐ and low‐risk groups in the TCGA dataset. The results revealed that pathways, such as ECM receptor interaction (NES = 2.143, normalized *p* = 0.002), focal adhesion (NES = 2.052, normalized *p* = 0.002), and adherence junction (NES = 1.605, normalized *p* = 0.032) etc. were enriched in high m^6^ARLncSig group, suggesting that m^6^ARLncRNAs played critical roles in the progression of NSCLC (Figure S5).

### 3.7 Independent prognostic assessment and clinical stratification analysis of m^6^ARLncSig

**FIGURE 6:**
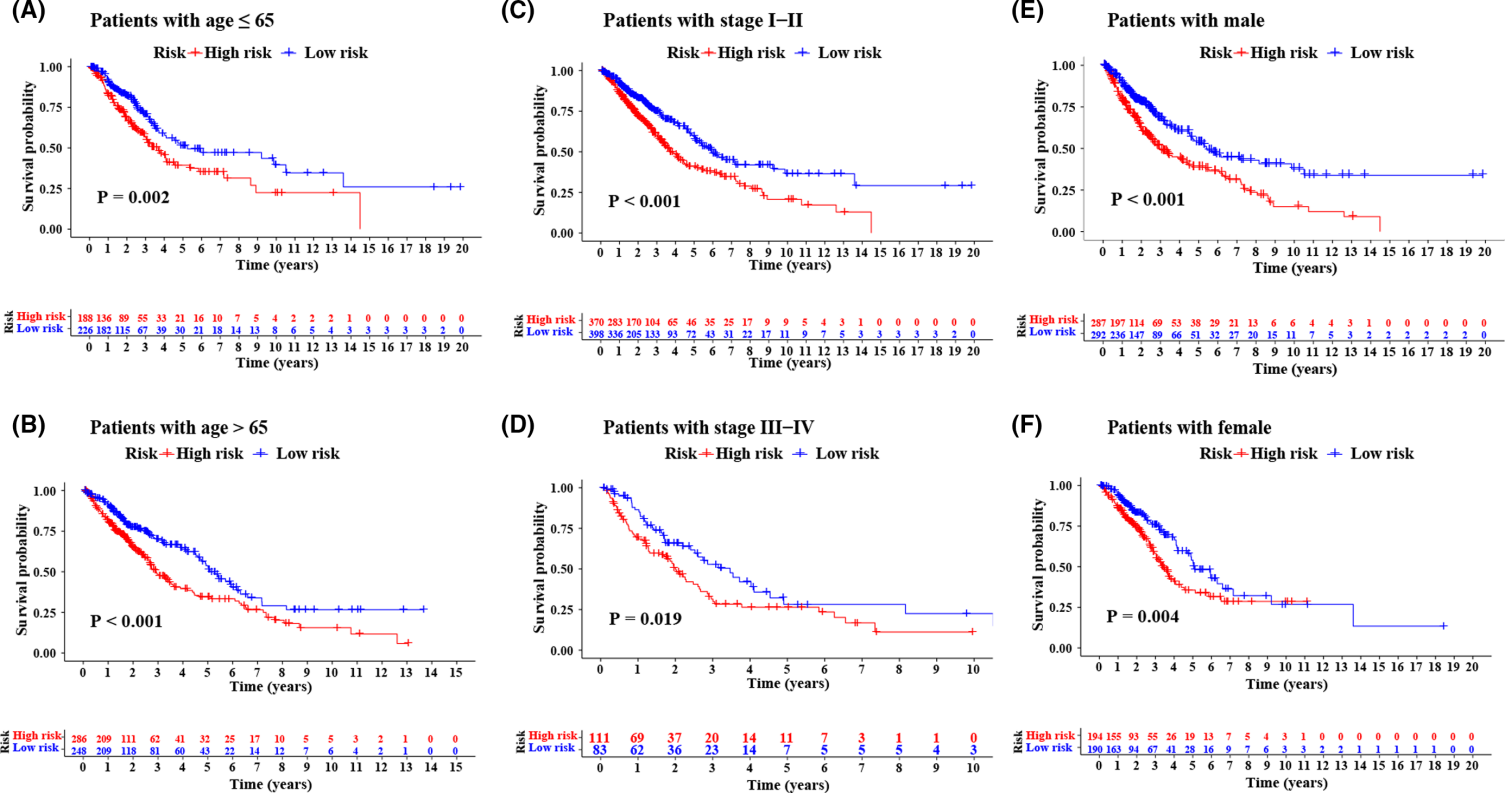
Clinical stratification analysis of the survival difference. (A–F) Kaplan–Meier survival curves showing the survival difference between the high‐ and low‐risk groups in multiple subgroups of NSCLC patients, including patients aged ≤65 or >65 (A–B), patients with tumor Stage I–II or III–IV (C–D), and patients with male or female (E–F), respectively.

Independent prognostic analysis revealed that m^6^ARLncSig was an independent risk factor of OS in NSCLC patients in the training dataset, further validated in the testing dataset, GSE37745, GSE31210, and GSE30219 (Table 2). We also conducted a clinical stratification analysis to assess the prognostic value of m^6^ARLncSig in various subgroups separated by age, gender, and tumor stage. The results suggested that regardless of age (Figure 6A–B), stage (Figure 6C–D), and sex (Figure 6E–F) in NSCLC, patients with high‐risk m^6^ARLncSig scores exhibited a worse prognosis.

**TABLE 2:** Univariate and multivariate Cox regression analysis of the m^6^ARLncSig and clinical features for the independent prognostic significance in five datasets

Variables	Univariable model				Multivariable model			
	HR	95% CI lower	95% CI Higher	*p‐*value	HR	95% CI lower	95% CI higher	*p‐*value
TCGA training dataset (*N* = 483)								
Age	1.015	0.999	1.031	0.062				
Gender	0.864	0.646	1.155	0.323				
Tumor stage	1.491	1.273	1.746	<0.001	1.390	1.179	1.640	<0.001
m^6^ARLncSig	1.260	1.195	1.328	<0.001	1.222	1.156	1.291	<0.001
TCGA testing dataset (*N* = 480)								
Age	1.002	0.986	1.019	0.801				
Gender	1.504	1.104	2.050	0.010	1.380	1.005	1.894	0.046
Tumor stage	1.530	1.316	1.778	<0.001	1.451	1.245	1.692	<0.001
m^6^ARLncSig	1.244	1.132	1.366	<0.001	1.212	1.103	1.332	<0.001
GSE37745 (*N* = 196)								
Age	1.025	1.006	1.045	0.010	1.024	1.004	1.044	0.016
Gender	1.096	0.789	1.523	0.585				
Tumor stage	1.270	1.049	1.539	0.014	1.281	1.054	1.557	0.013
m^6^ARLncSig	1.699	1.297	2.226	<0.001	1.626	1.252	2.113	<0.001
GSE31210 (*N* = 226)								
Age	1.025	0.977	1.075	0.306				
Gender	1.519	0.780	2.955	0.219				
Tumor stage	4.232	2.175	8.236	<0.001	3.153	1.580	6.291	0.001
m^6^ARLncSig	1.007	1.004	1.010	<0.001	1.006	1.002	1.009	0.001
GSE30219 (*N* = 268)								
Age	1.038	1.023	1.054	<0.001	1.037	1.022	1.053	<0.001
Gender	1.646	1.033	2.622	0.036	1.300	0.810	2.085	0.2770
Tumor stage	1.690	1.424	2.007	<0.001	1.692	1.416	2.023	<0.001
m^6^ARLncSig	1.732	1.366	2.196	<0.001	1.396	1.101	1.769	0.006

Abbreviations: CI, confidence interval; HR, hazard ratio.

### 3.8 Relationship between m^6^ARLncSig and tumor‐immune infiltrating, TMB, and therapeutic sensitivity

We estimated the difference in tumor‐immune infiltrating cells between high‐ and low‐risk groups. The results indicated that the m^6^ARLncSig score was positively correlated with the infiltration level of the neutrophils, macrophages M0, and activated memory CD4 T cells (Figure S6A–C). And a significant negative correlation was observed in monocytes and naive B cells (Figure S6D–E). In addition, TMB was also significantly higher in the high‐ group than low‐ risk group (Figure S6F). We also found that T cell functions exhibited distinct changes in the two groups (Figure S6G). Furthermore, significant differences at the critical immune checkpoints were observed between the two risk groups (Figure S6H). These results indicated that m^6^ARLncSig was associated with the immune regulation of NSCLC patients.

**FIGURE 7:**
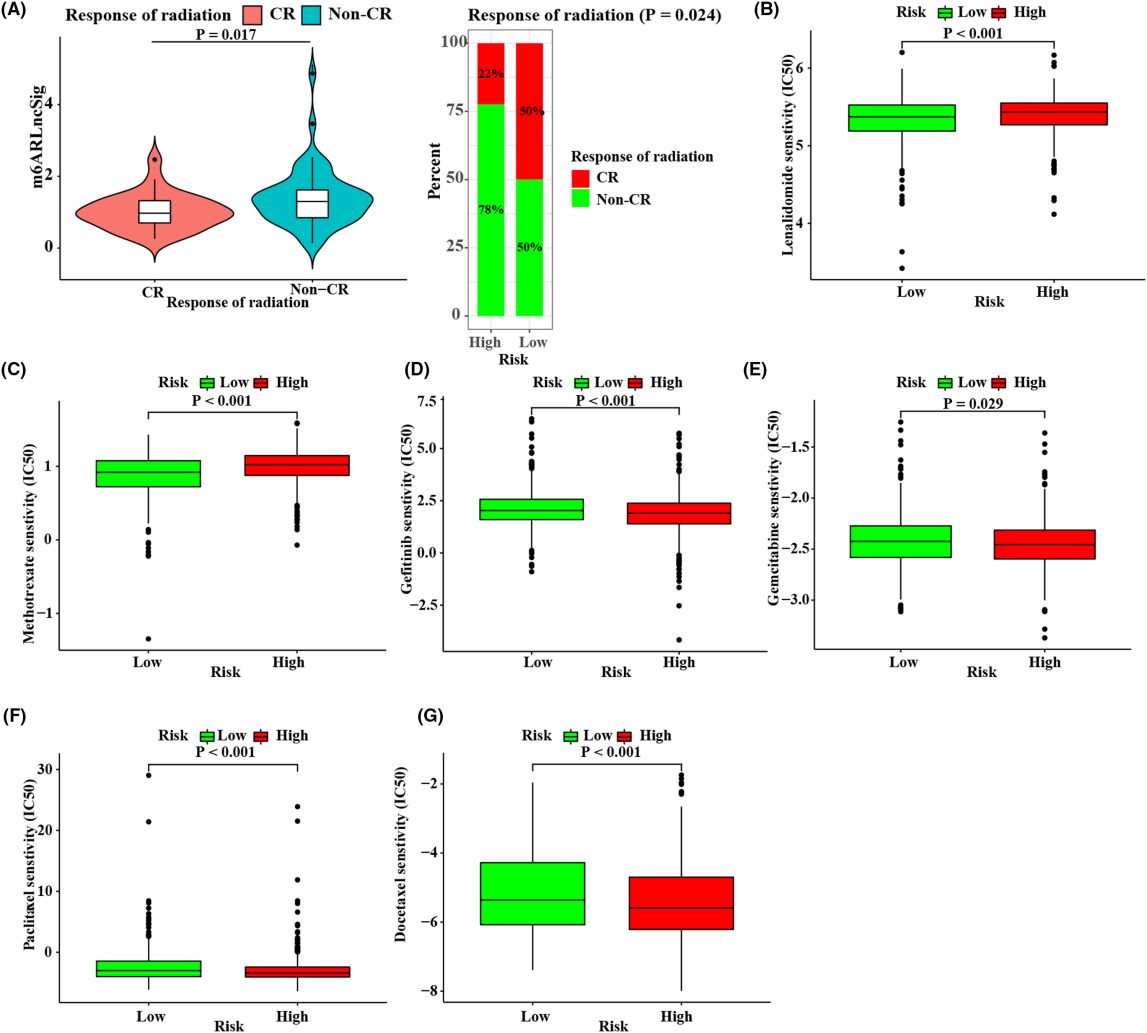
Relationships between m^6^ARLncSig and therapeutic effect. (A) The boxplots of the proportion of patients with NSCLC receiving radiotherapy between the high‐ and low‐risk groups in the training set. (B–G) The distribution of the estimated IC50 of lenalidomide (B), methotrexate (C), gefitinib (D), gemcitabine (E), paclitaxel (F), and docetaxel (G) in NSCLC patients.

Then, we examined whether an association existed between m^6^ARLncSig and radiotherapeutic response and sensitivity of common therapeutic drugs administered in NSCLC. The result showed that most patients with high m^6^ARLncSig did not respond or respond poorly to radiotherapy relative to low‐risk group (Figure 7A). And m^6^ARLncSig was positively associated with half inhibitory concentration (IC50) of drugs, such as lenalidomide and methotrexate (Figure 7B–C). On the contrary, the m^6^ARLncSig score was negatively correlated with IC50 of gefitinib, gemcitabine, paclitaxel, and docetaxel, which suggested that the signature had an excellent potential for drug‐sensitivity prediction (Figure 7D–G).

### 3.9 Construction and validation of a nomogram

**FIGURE 8:**
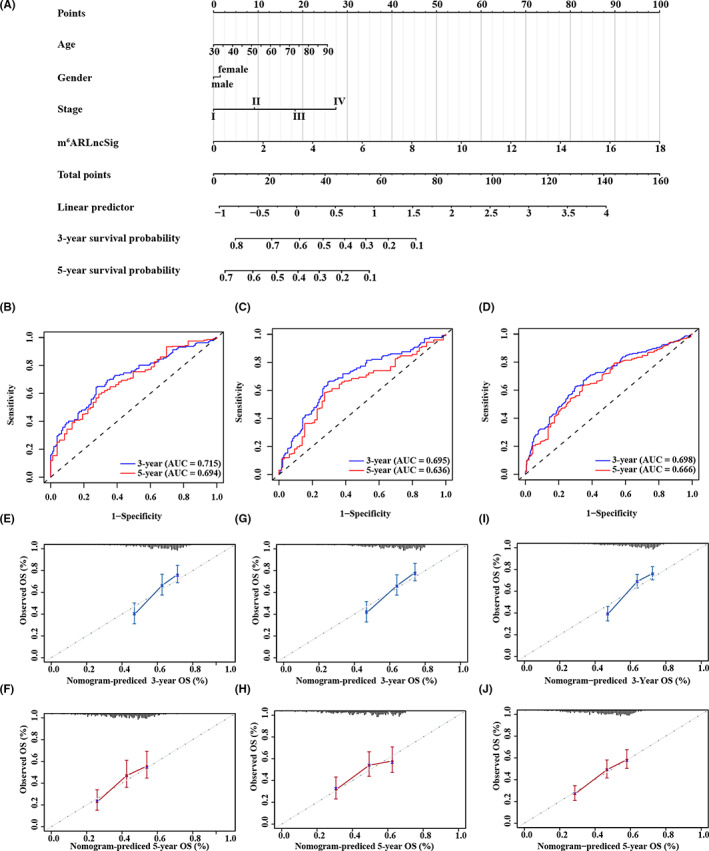
Construction and evaluation of a nomogram for survival prediction of NSCLC patients. (A) The nomogram was developed in the training dataset for predicting the 3‐ and 5‐year survival of NSCLC patients. (B–D) ROC curves for 3‐ and 5‐year survival prediction of the nomogram in the training dataset (B), the testing dataset (C), and the entire TCGA dataset (D), respectively. (E–J) The calibration plots of the training dataset (E–F), testing dataset (G–H), and whole TCGA dataset (I–J) in the 3‐ and 5‐year survival, respectively.

To improve the m^6^ARLncSig's clinical practicability for clinical survival prediction of NSCLC, we established a nomogram scoring system in the training dataset (Figure 8A). Our results showed the improved performance of survival prediction with a C‐index of 0.66 and AUCs of ROC of 0.715 and 0.694 for 3‐ and 5‐year survival predictions, respectively (Figure 8B). The findings were validated in the testing and whole TCGA datasets with a C‐index of 0.671 and 0.666. The AUCs of ROC for 3‐ and 5‐year survival predictions were 0.695 and 0.636 in the testing dataset and 0.698 and 0.666 in the entire TCGA dataset (Figure 8C–D). Calibration plots showed excellent consistency between the observed and predicted values for 3‐ and 5‐year OS prediction in the testing, training, and the entire TCGA dataset (Figure 8E–J). Therefore, these findings indicated that the nomogram possessed an excellent prospect of clinical application for prognosis evaluation of NSCLC.

## 4 DISCUSSION

The therapeutic strategy and prognosis of NSCLC traditionally depend on the clinical factors, such as TNM stage and histologic grade.^24^, ^25^ However, the prediction of survival within the same TNM staging varies widely due to tumor heterogeneity, population diversity, and complicated carcinogenic mechanisms. Therefore, identifying novel molecular biomarkers for estimating the prognosis and effective treatment of NSCLC patients is significant.

Accumulating evidence has demonstrated that aberration m^6^A modification is dramatically associated with multiple cancer pathogenesis.^26^ Notably, m^6^A peaks are present in approximately 67% lncRNAs of 3′ UTRs.^8^, ^27^ m^6^A methylation has been reported to act as a lncRNA structural switch, participate in the lncRNA‐mediated ceRNA modulation, and enhance the stability of lncRNA, thereby influencing cancer development and progression.^14^ For instance, METTL3‐induced lncRNA ABHD11‐AS1 was closely correlated with the unfavorable prognosis of NSCLC patients.^28^ Therefore, looking into the interplay between m^6^A modifications and lncRNAs in NSCLC is of paramount importance.

In the present study, we identified 491 m^6^A‐related lncRNAs (m^6^ARLncRNAs) in 1835 NSCLC patients and explored their prognostic implication and clinical relevance in NSCLC. The m^6^ARLncRNAs could divide patients into two distinct molecular clustering patterns, which showed significantly different survival outcomes. These findings prompted us to assume whether m^6^ARLncRNAs might be a prognostic predictor for NSCLC patients. Therefore, we constructed the prognostic m^6^ARLncRNAs signature (m^6^ARLncSig). Patients in the high‐ and low‐risk groups divided in terms of m^6^ARLncSig scores exhibited remarkedly different survival outcomes. In addition, we found that the m^6^ARLncSig scores were closely associated with clinical characteristics of NSCLC patients and m^6^ARLncSig could independently predict the prognosis of NSCLC patients. In addition, we conducted a GSEA analysis and found multiple KEGG pathways were linked with the tumorigenesis and progression of NSCLC. It has been reported that cell adhesion plays an essential role in critical biological processes, including cell motility, proliferation, differentiation, and survival, which might explain the poor survival in the high‐risk group.

It is generally believed that the response to chemotherapy and radiotherapy may vary with patients and only a small subset of NSCLC patients respond well. Early assessment of treatment efficacy in predictive biomarkers is crucial for NSCLC patients. In the study, we found that patients with high m^6^ARLncSig tended to respond poorly to radiotherapy and common therapeutics drugs, including gemcitabine, paclitaxel, docetaxel, and gefitinib in the high‐risk group. Our finding can, to some extent, help clinicians individualize treatment for different NSCLC patients based on the m^6^ARLncSig model. TMB is considered a promising biomarker for evaluating immunotherapy's efficacy, and the patients with high TMB could gain favorable survival outcomes from immunotherapy.^29^ In the present study, we found that NSCLC patients with high m^6^ARLncSig scores displayed conspicuously higher TMB. The result suggested that patients in the high‐risk group were more likely to benefit from immunotherapy. The immune infiltrates in the tumor environment are increasingly reported to be associated with the prognosis of NSCLC patients.^30^ In our study, we found that monocytes and naïve B cells were higher in the low‐risk group and associated with more favorable survival outcomes. Cluster 1 with poor worse had more elevated neutrophils, which was considered anti‐inflammatory. Furthermore, the expression level of neutrophils was positively correlated with the m^6^ARLncSig score.

Many tumor‐related lncRNAs have been studied previously. Among the 12 m^6^ARLncRNAs, LINC01138, SNHG12, ITGA9‐AS1, and TSPOAP1‐AS1 were involved in malignant phenotypes of cancers. For example, upregulated LINC01138 promoted cell growth and was considered a prognostic indicator in hepatocellular carcinoma,^31^ clear cell renal cell carcinoma,^32^ and gastric cancer.^33^ These findings were consistent with our study about the role of LINC01138 in NSCLC. Knockdown of SNHG12 suppressed metastasis, epithelial‐mesenchymal transition, and mediated doxorubicin resistance of osteosarcoma.^34^, ^35^ Overexpressed SNHG12 in LUAD promoted tumor proliferation and metastasis.^36^ However, in our study, SNHG12 was a protective factor. This inconsistency might be ascribed to the normal samples being excluded, whose OS data were not available in the TCGA dataset. Zhang et al. revealed that ITGA9‐AS1 was downregulated and positively correlated with the survival probability in breast cancer.^37^ In the present study, we identified that overexpressing TSPOAP1‐AS1 had a better prognosis in NSCLC patients, which was in agreement with previous studies in pancreatic ductal adenocarcinoma and LUAD.^38^, ^39^ The role of the remaining prognostic m^6^ARLncRNAs was the first to be studies in NSCLC and reports about how the m^6^ARLncRNAs interacted with m^6^A regulators have been scanty. Therefore, further experimental confirmation is warranted to fully understand the functional role of prognostic lncRNAs in NSCLC both in vitro and in vivo.

Several prior studies have also constructed the prognostic models by using similar methods. For example, Jiang et al. built an m6A‐related gene signature for prognostic evaluation in LUAD using principal component analysis (PCA).^21^ Guo et al. examined the prognostic role of genome instability‐derived lncRNAs in LUAD.^22^ Weng et al. and Zheng et al. looked into the prognostic prediction of m^6^ARLncRNAs in 594 LUAD patients and 549 LUSC patients, respectively.^16^, ^17^ However, our model outperformed their models in terms of AUCs in the ROC curve for one‐year survival prediction. Moreover, these models were constructed only in LUAD or LUSC subtype and have limited analyzing samples. Our study constructed and validated a prognostic m^6^ARLncSig in 1835 NSCLC patients and further verified it in our cohorts.

Our study also had some limitations. Firstly, the constructed m^6^ARLncSig possessed excellent prognosis and drug‐sensitivity predictive potential. However, this in‐silico evidence needs further validation. Additionally, the m^6^A modification of lncRNAs is a rather complicated process, and future research is focused on confirming the interaction between lncRNAs and m6A modification in vitro and in vivo.

In conclusion, we, for the first time, constructed the m^6^ARLncSig model in NSCLC patients, which was highly associated with clinical features of NSCLC and could independently predict patients' prognosis. We also demonstrated that the m^6^ARLncSig score could predict the radiotherapeutic response and chemotherapeutic sensitivity of NSCLC patients. These results suggest that m^6^ARLncSig could be used as a new potential and promising prognosis indicator and provide an individual treatment strategy for NSCLC.

## AUTHOR CONTRIBUTION

Yang Jin, Wenjing Xiao, and Wei Geng contributed to the conception and design of the study. Yang Jin organized the database. Wenjing Xiao and Wei Geng performed the statistical analysis and wrote the first draft of the manuscript. Juanjuan Xu, Qi Huang, Jinshuo Fan, Qi Tan, Zhengrong Yin, and Yumei Li provided comments during the writing. Guanghai Yang provided help in the collection of clinical tissues. All authors contributed to manuscript revision, read, and approved the submitted version.

## CONFLICT OF INTEREST

The authors declare that they have no competing interests.

## ETHICS STATEMENT

The study was approved by Tongji Medical College, Huazhong University of Science and Technology (protocol: 2010‐S202) and all patients provided informed consent.

## Supporting information


Figure S1

Figure S2

Figure S3

Figure S4

Figure S5

Figure S6

Table S1

Table S2

Table S3

Table S4

## Data Availability

The data supporting this study's findings are available from the corresponding author upon reasonable request.
