# Supplementary figures and images for: Construction and validation of a nomogram based on N6‐Methylandenosine‐related lncRNAs for predicting the prognosis of non‐small cell lung cancer patients
Source: Cancer Med. 2022 Jun 21;12(2):2058–74. doi: 10.1002/cam4.4961 (PMC9883402; doi:10.1002/cam4.4961)

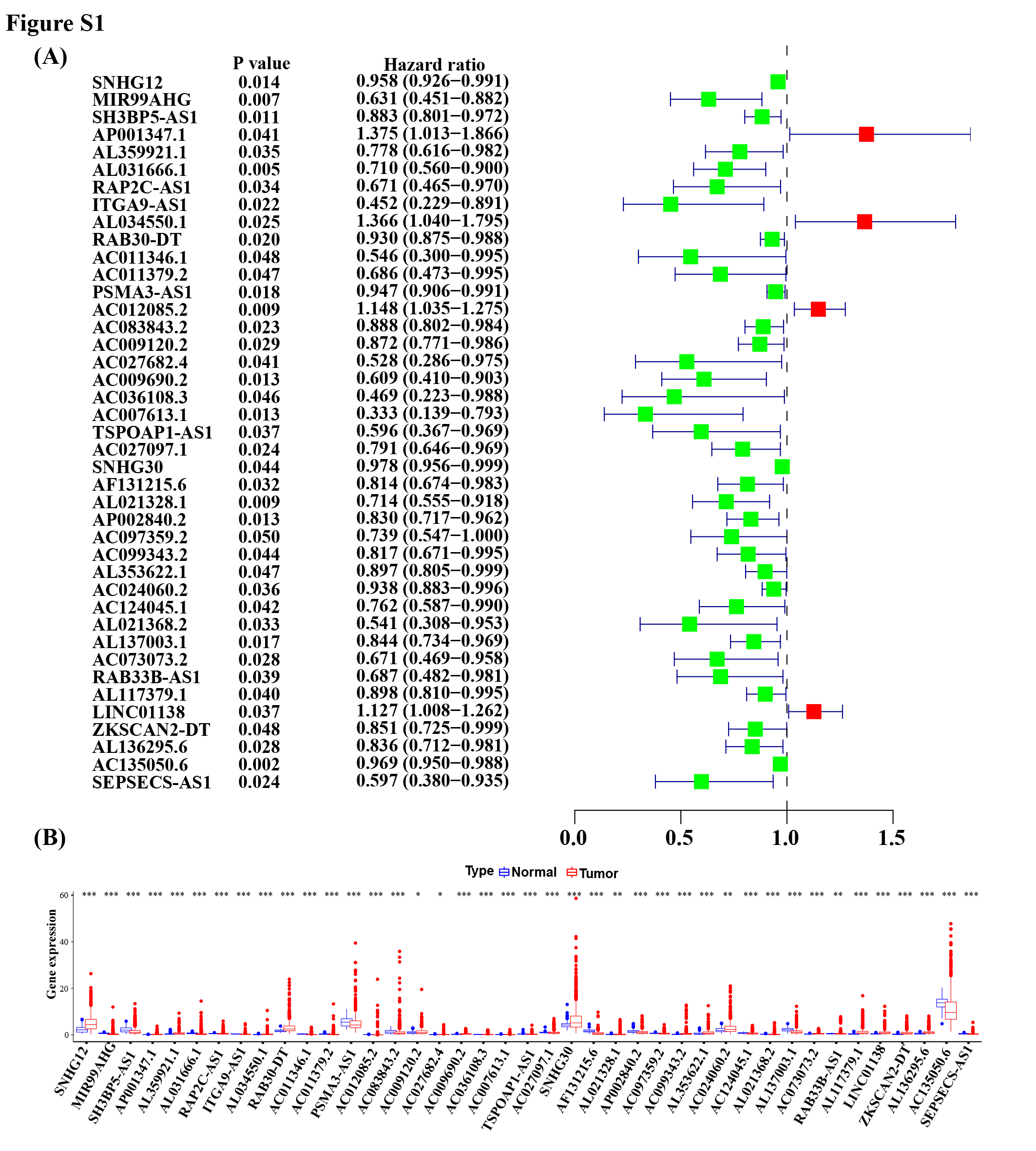

Supplement: Supplementary file 1 — Figure S1 [file CAM4-12-2058-s002.tif]

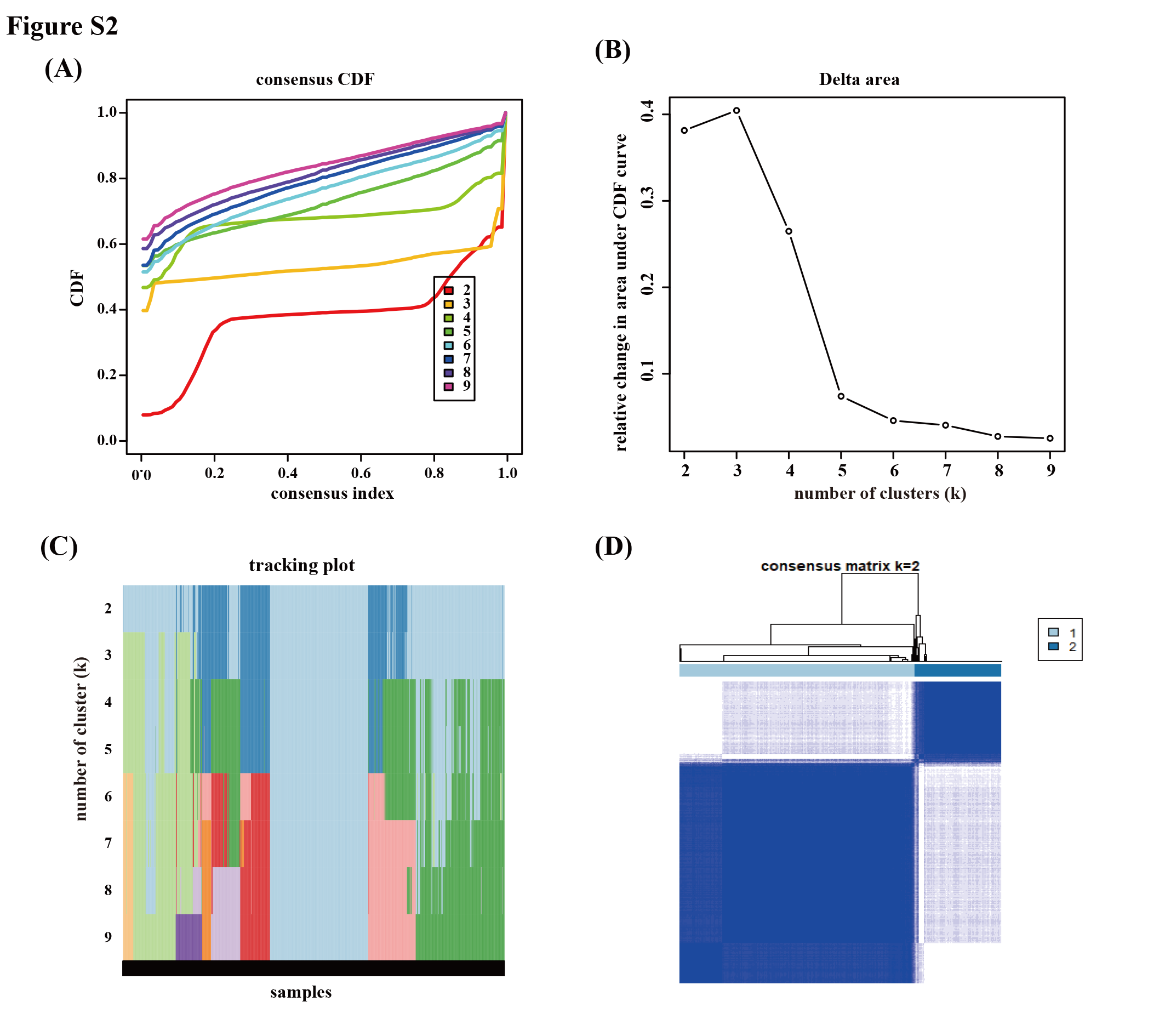

Supplement: Supplementary file 2 — Figure S2 [file CAM4-12-2058-s006.tif]

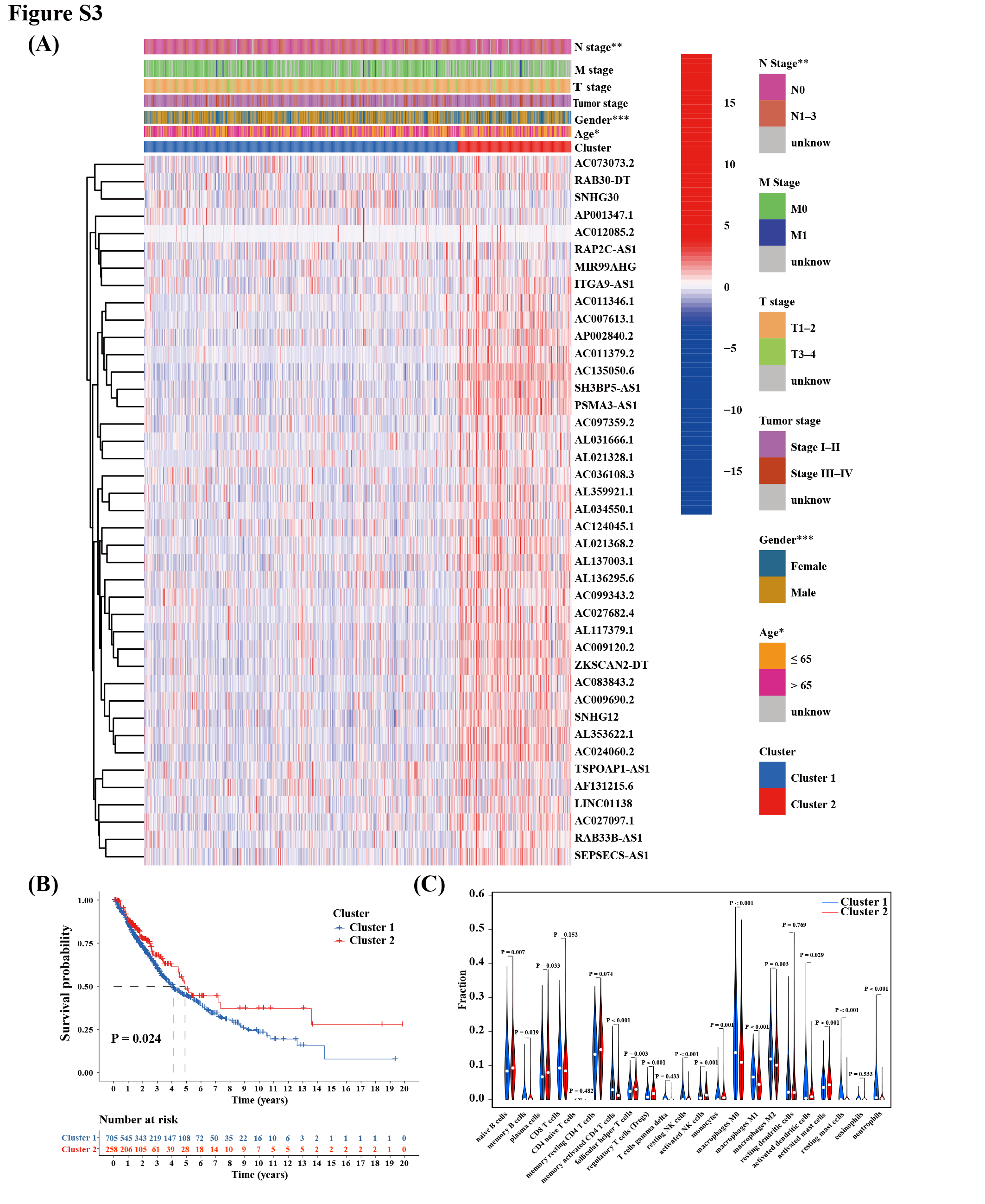

Supplement: Supplementary file 3 — Figure S3 [file CAM4-12-2058-s008.tif]

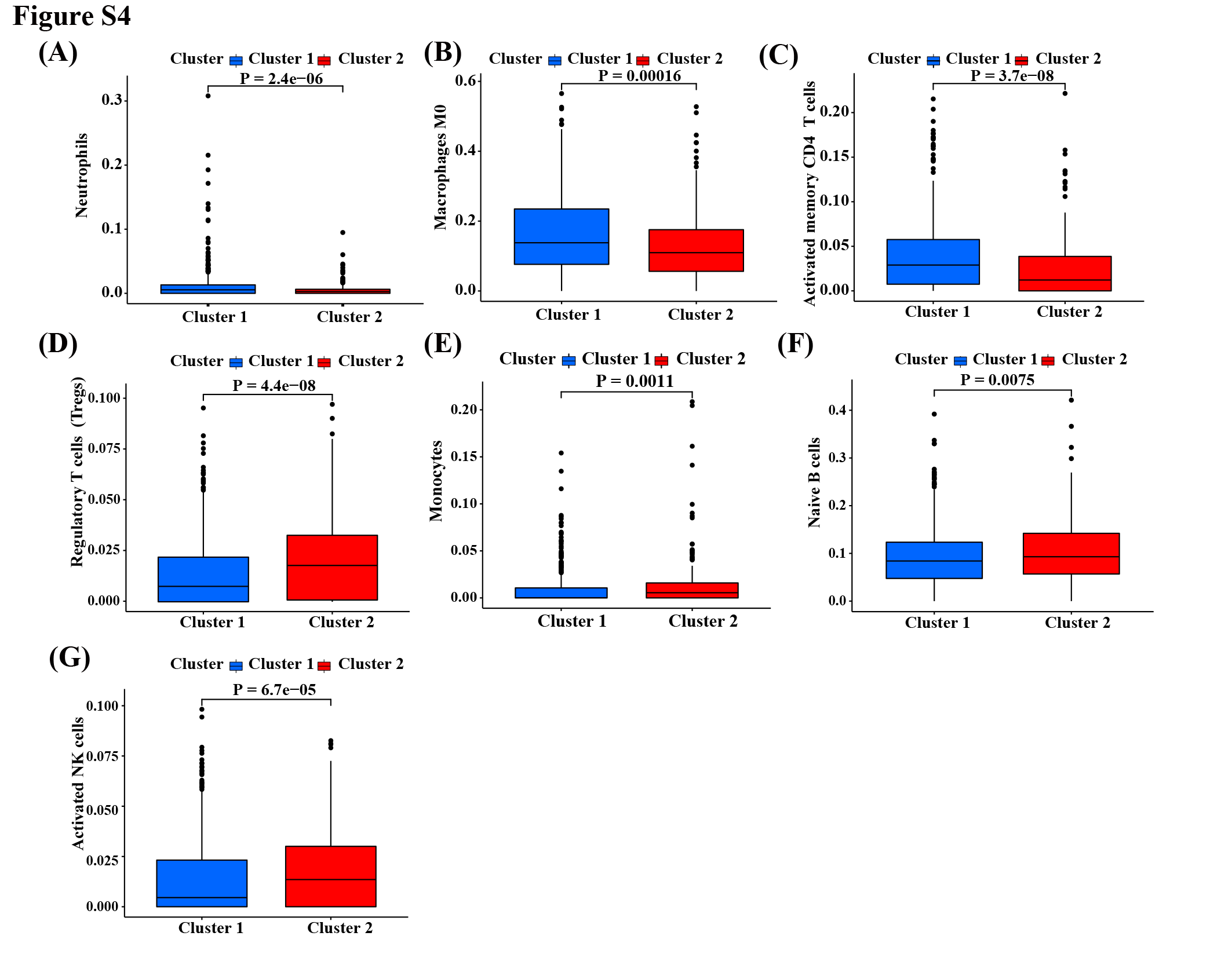

Supplement: Supplementary file 4 — Figure S4 [file CAM4-12-2058-s010.tif]

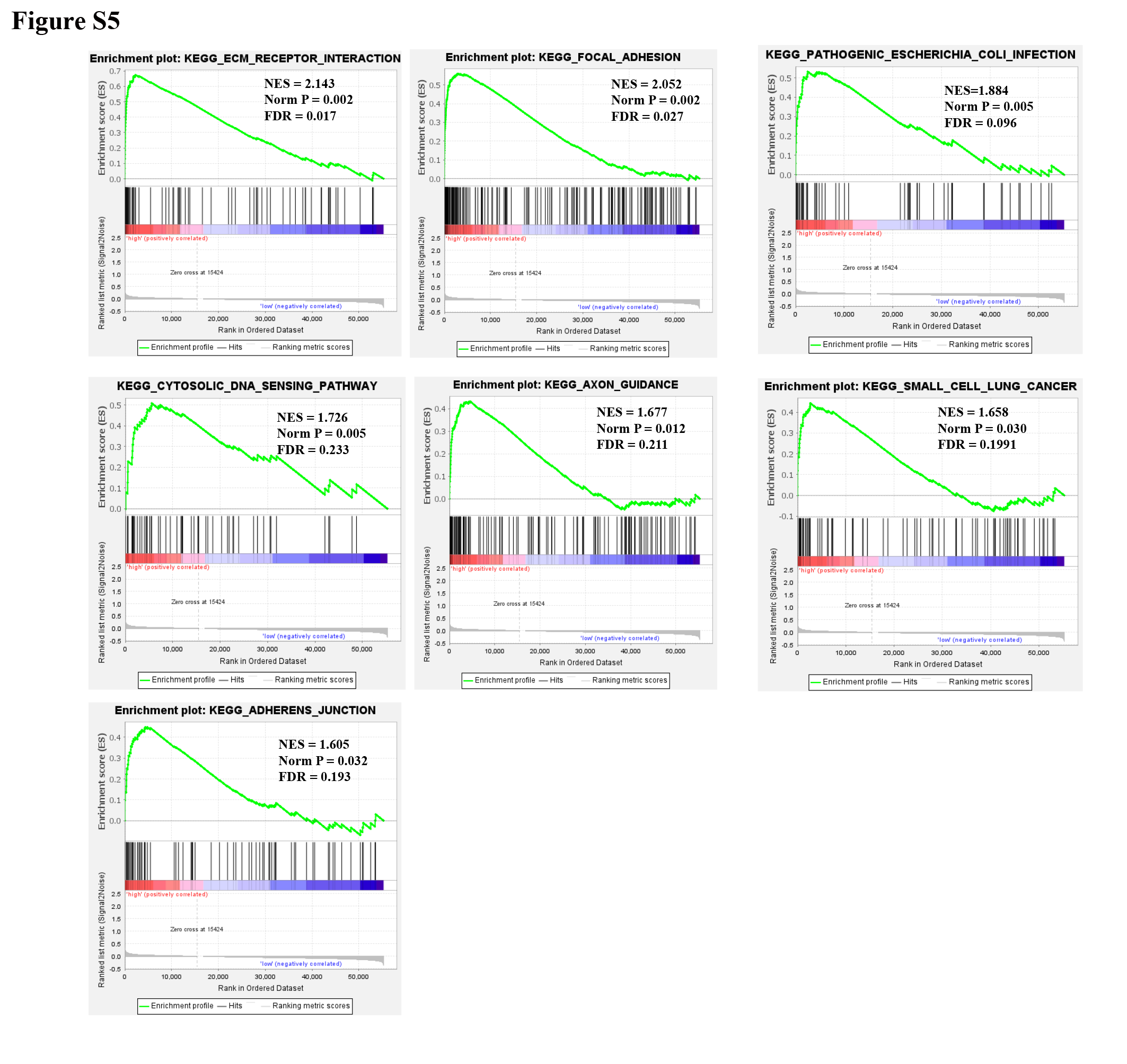

Supplement: Supplementary file 5 — Figure S5 [file CAM4-12-2058-s003.tif]

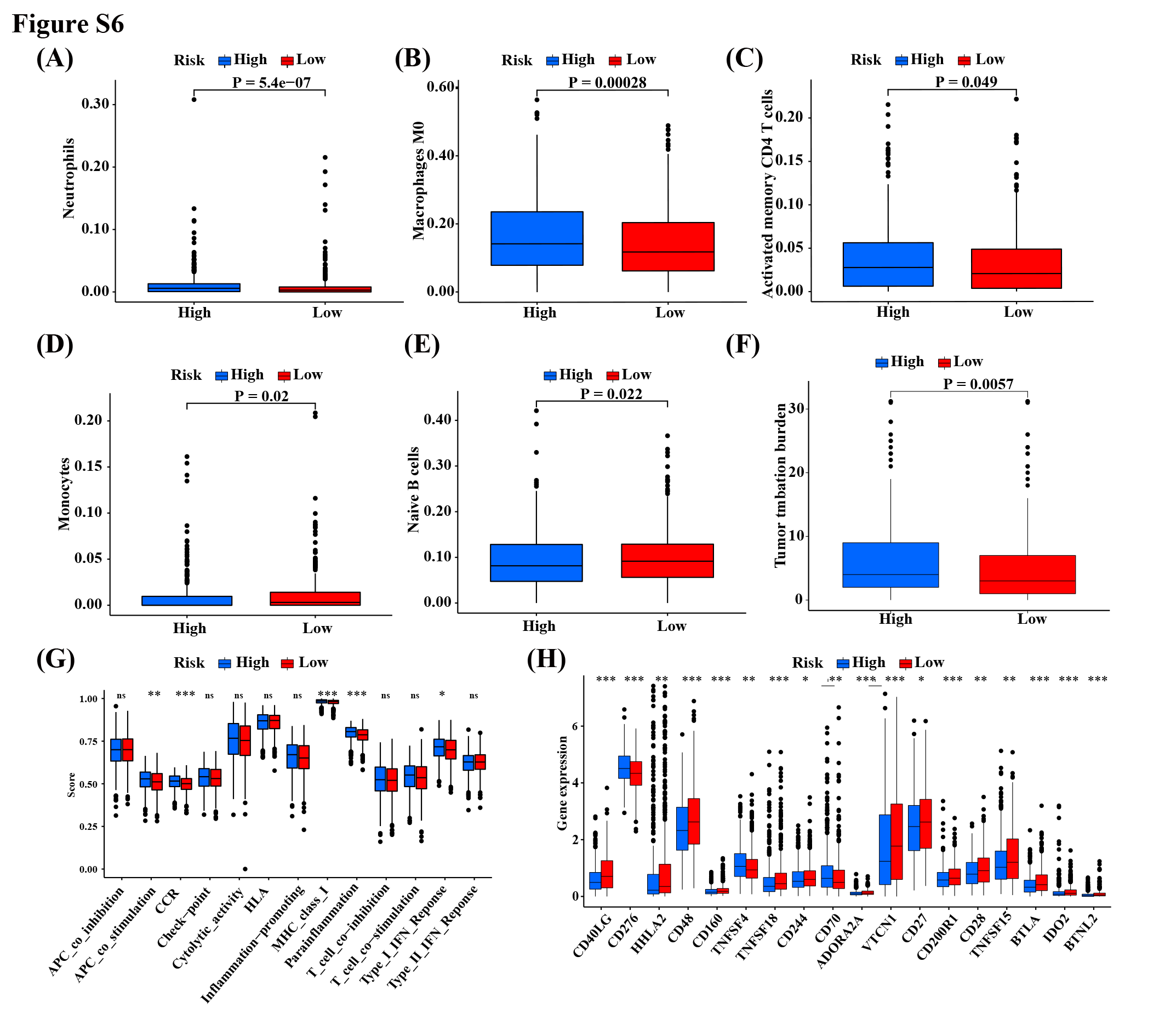

Supplement: Supplementary file 6 — Figure S6 [file CAM4-12-2058-s004.tif]
