# Supplementary material for: Construction and validation of a nomogram based on N6‐Methylandenosine‐related lncRNAs for predicting the prognosis of non‐small cell lung cancer patients
Source: Cancer Med. 2022 Jun 21;12(2):2058–74. doi: 10.1002/cam4.4961 (PMC9883402; doi:10.1002/cam4.4961)
Supplement: Supplementary file 7 — Table S1 [file CAM4-12-2058-s007.docx]

**Table S1. Primer sets used for qRT-PCR**

|  | **Primers** | **Sequence** |
| --- | --- | --- |
| SNHG30 | Forward | 5’-TGCTCAGGGACAGGTTATTGG-3' |
|  | Reverse | 5’-CGGGAAGACTGAGAGTGAGG-3’ |
| AL021328.1 | Forward | 5’-CAGACCCGACCTCTTCCCTC-3’ |
|  | Reverse | 5’-CCTCCTCGCTTTCTCTCCCA-3’ |
| AC024060.2 | Forward | 5’-TTTTACTTTACCATGCTCAT-3’ |
|  | Reverse | 5’-AGACTGTATTTCTGTTTTCC-3’ |
| AL137003.1 | Forward | 5’-CACAAATAAGGCTGGGGAGA-3’ |
|  | Reverse | 5’-TTGGTGGAGGGTCAAGAAAG-3’ |
| LINC01138 | Forward | 5’-GACTGTGTCATACTTCCCAT-3’ |
|  | Reverse | 5’-TTTTGTTGTTCCAACTGCTC-3’ |
| SEPSECS-AS1 | Forward | 5’-TAAGAATACAACCACGAAAAACA-3’ |
|  | Reverse | 5’-CAAACTGAGAATCAAATCAATAA-3’ |
| AL034550.1 | Forward | 5’-TGGGCTGCTTGGAGATGGGC-3’ |
|  | Reverse | 5’-CAGGTGGGGTGGGATGGGGG-3’ |
| ITGA9-AS1 | Forward | 5’-TTTCTCCATTTTCTGTCTTCA-3’ |
|  | Reverse | 5’-TAGGCAACCGCTAATCTACTT-3’ |
| AP001347.1 | Forward | 5’-GATAAAGCAGATGAAAATAG-3’ |
|  | Reverse | 5’-ATCAAAGAAAAAATACACAC-3’ |
| AC083843.2 | Forward | 5’-GATGGGGAGGAAAGGGATTA-3’ |
|  | Reverse | 5’-GACACGACAGGGCTTGGAGA-3’ |
| SNHG12 | Forward | 5’-GGACCTATGGAGTTGGGACAAT-3’ |
|  | Reverse | 5’-AAGTTCAGTAGCACACTGCATAA-3’ |
| TSPOAP1-AS1 | Forward | 5’- TCGCCGATGGGAAGAACAAC-3’ |
|  | Reverse | 5’- AAGAGGAAGTCTAAGCGCCG-3’ |
| GAPDH | Forward | 5'-GGGCTGCTTTTAACTCTGGT-3' |
|  | Reverse | 5'-TGATTTTGGAGGGATCTCGC-3' |
