# Supplementary material for: Construction and validation of a nomogram based on N6‐Methylandenosine‐related lncRNAs for predicting the prognosis of non‐small cell lung cancer patients
Source: Cancer Med. 2022 Jun 21;12(2):2058–74. doi: 10.1002/cam4.4961 (PMC9883402; doi:10.1002/cam4.4961)
Supplement: Supplementary file 8 — Table S2 [file CAM4-12-2058-s009.docx]

**Table S2. The clinicopathological features of the NSCLC samples**

| **Sample** | **Sex** | **Age** | **Tumor stage** | **Survival time (d)** | **status (dead=1, alive=0)** | | **Histological**  **subtype** |
| --- | --- | --- | --- | --- | --- | --- | --- |
| 1  2  3  4  5  6  7  8  9  10  11  12  13  14  15  16  17  18  19  20  21  22  23  24  25  26  27  28  29  30  31  32  33  34  35  36  37  38  39  40  41  42  43  44  45  46 | male  male  female  female  male  female  female  male  female  male  male  male  male  female  female  male  male  male  male  male  female  male  female  female  female  male  male  male  female  female  female  male  female  male  male  male  female  male  male  male  male  male  male  male  female  male | 61  65  59  51  53  52  36  70  64  64  54  55  57  63  58  43  55  68  56  62  61  54  56  74  56  63  58  55  60  69  67  50  62  56  46  53  50  58  59  60  75  68  61  60  46  65 | IB  IB  IB  IIIA  IA2  IIIB  IA3  IB  IB  IA2  IIIA  IA2  IIIA  IIIA  IIIA  IIB  IIB  IB  IB  IB  IB  IA2  IV  IIIA  IB  IIIA  IIB  IIIA  IA3  IB  IA3  IIIA  IIIB  IV  IIIA  IB  IIIB  IA2  IIIA  IB  IB  IB  IB  IIA  IIIA  1A3 | 1680  1427  2084  830  1632  1586  527  569  1485  2414  741  842  613  1536  801  830  836  885  1578  1649  1507  1565  2523  251  792  861  539  383  564  1570  836  714  792  339  660  673  806  1537  655  808  465  464  1296  561  436  1109 | 0  1  0  0  0  0  0  0  0  0  1  0  1  0  0  0  0  0  1  0  0  0  1  1  1  1  0  1  0  0  0  0  0  1  1  0  0  0  0  0  0  0  0  0  0  0 | LUAD  LUAD  LUAD  LUAD  LUAD  LUAD  LUAD  LUAD  LUAD  LUAD  LUAD  LUAD  LUAD  LUAD  LUAD  LUAD  LUAD  LUAD  LUSC  LUAD  LUAD  LUAD  LUAD  LUAD  LUAD  LUSC  LUAD  LUAD  LUAD  LUAD  LUAD  LUAD  LUSC  LUAD  LUAD  LUAD  LUAD  LUAD  LUSC  LUAD  LUAD  LUAD  LUAD  LUAD  LUAD  LUAD | |

LUAD: lung adenocarcinoma; LUSC: lung squamous cell carcinoma.
