# Supplementary material for: Construction and validation of a nomogram based on N6‐Methylandenosine‐related lncRNAs for predicting the prognosis of non‐small cell lung cancer patients
Source: Cancer Med. 2022 Jun 21;12(2):2058–74. doi: 10.1002/cam4.4961 (PMC9883402; doi:10.1002/cam4.4961)
Supplement: Supplementary file 9 — Table S3 [file CAM4-12-2058-s005.docx]

**Table S3. Correlations between the** **m^6^ARLncRNAs and 23 m^6^A regulators in the TCGA dataset with the condition (|Pearson R| > 0.4 and *P* < 0.001)**

| **m^6^A** | **lncRNA** | **Cor** | ***P*-value** | **Regulation** |
| --- | --- | --- | --- | --- |
| RBM15 | AC007613.1 | 0.490441 | 7.34E-64 | positive |
| METTL3 | AC009120.2 | 0.421945 | 5.05E-46 | positive |
| RBM15 | AC009120.2 | 0.416138 | 1.09E-44 | positive |
| YTHDC1 | AC009120.2 | 0.402007 | 1.48E-41 | positive |
| YTHDC2 | AC009120.2 | 0.619613 | 5.48E-111 | positive |
| METTL3 | AC009690.2 | 0.452041 | 2.31E-53 | positive |
| RBM15 | AC009690.2 | 0.495599 | 2.25E-65 | postive |
| YTHDC2 | AC009690.2 | 0.528224 | 1.40E-75 | postive |
| YTHDC2 | AC011346.1 | 0.410599 | 1.92E-43 | postive |
| YTHDC2 | AC011379.2 | 0.409524 | 3.33E-43 | postive |
| RBM15 | AC012085.2 | 0.508281 | 3.27E-69 | postive |
| YTHDC2 | AC012085.2 | 0.450825 | 4.73E-53 | postive |
| METTL3 | AC024060.2 | 0.421386 | 6.80E-46 | postive |
| RBM15 | AC024060.2 | 0.430742 | 4.30E-48 | postive |
| YTHDC2 | AC024060.2 | 0.598865 | 6.19E-102 | postive |
| RBM15 | AC027097.1 | 0.460343 | 1.61E-55 | postive |
| YTHDC2 | AC027097.1 | 0.491961 | 2.64E-64 | postive |
| FMR1 | AC027682.4 | 0.407121 | 1.13E-42 | postive |
| RBM15 | AC027682.4 | 0.626474 | 3.92E-114 | postive |
| YTHDC2 | AC027682.4 | 0.605974 | 5.82E-105 | postive |
| RBM15 | AC036108.3 | 0.577888 | 1.97E-93 | postive |
| YTHDC2 | AC036108.3 | 0.586611 | 6.83E-97 | postive |
| RBM15 | AC073073.2 | 0.447414 | 3.47E-52 | postive |
| FMR1 | AC083843.2 | 0.433346 | 1.02E-48 | postive |
| RBM15 | AC083843.2 | 0.65199 | 1.49E-126 | postive |
| YTHDC2 | AC083843.2 | 0.589251 | 5.84E-98 | postive |
| RBM15 | AC097359.2 | 0.429649 | 7.84E-48 | postive |
| FMR1 | AC099343.2 | 0.428365 | 1.58E-47 | postive |
| RBM15 | AC099343.2 | 0.640158 | 1.20E-120 | postive |
| YTHDC2 | AC099343.2 | 0.631596 | 1.56E-116 | postive |
| YTHDC2 | AC124045.1 | 0.460897 | 1.15E-55 | postive |
| METTL3 | AC135050.6 | 0.40671 | 1.39E-42 | postive |
| YTHDC2 | AF131215.6 | 0.452548 | 1.71E-53 | postive |
| RBM15 | AL021328.1 | 0.475854 | 1.03E-59 | postive |
| YTHDC2 | AL021328.1 | 0.449545 | 1.00E-52 | postive |
| FMR1 | AL021368.2 | 0.418373 | 3.36E-45 | postive |
| METTL14 | AL021368.2 | 0.434325 | 5.93E-49 | postive |
| RBM15 | AL021368.2 | 0.570621 | 1.26E-90 | postive |
| YTHDC2 | AL021368.2 | 0.671516 | 6.65E-137 | postive |
| RBM15 | AL031666.1 | 0.633951 | 1.19E-117 | postive |
| YTHDC2 | AL031666.1 | 0.508694 | 2.44E-69 | postive |
| RBM15 | AL034550.1 | 0.496784 | 9.99E-66 | postive |
| YTHDC2 | AL034550.1 | 0.466558 | 3.56E-57 | postive |
| METTL3 | AL117379.1 | 0.43366 | 8.59E-49 | postive |
| RBM15 | AL117379.1 | 0.490273 | 8.22E-64 | postive |
| YTHDC2 | AL117379.1 | 0.493855 | 7.35E-65 | postive |
| METTL3 | AL136295.6 | 0.534092 | 1.55E-77 | postive |
| RBM15 | AL136295.6 | 0.42905 | 1.09E-47 | postive |
| METTL14 | AL137003.1 | 0.407167 | 1.11E-42 | postive |
| YTHDC2 | AL137003.1 | 0.557977 | 6.53E-86 | postive |
| METTL3 | AL353622.1 | 0.413001 | 5.56E-44 | postive |
| FMR1 | AL359921.1 | 0.418903 | 2.54E-45 | postive |
| METTL3 | AL359921.1 | 0.429006 | 1.11E-47 | postive |
| RBM15 | AL359921.1 | 0.572194 | 3.15E-91 | postive |
| YTHDC2 | AL359921.1 | 0.57548 | 1.71E-92 | postive |
| RBMX | AP001347.1 | 0.402921 | 9.40E-42 | postive |
| YTHDC2 | AP002840.2 | 0.451684 | 2.85E-53 | postive |
| RBM15 | ITGA9-AS1 | 0.458548 | 4.76E-55 | postive |
| YTHDC2 | ITGA9-AS1 | 0.463557 | 2.26E-56 | postive |
| FMR1 | LINC01138 | 0.402462 | 1.18E-41 | postive |
| METTL14 | MIR99AHG | 0.408429 | 5.82E-43 | postive |
| RBM15 | MIR99AHG | 0.482155 | 1.76E-61 | postive |
| YTHDC2 | MIR99AHG | 0.513934 | 5.66E-71 | postive |
| FMR1 | PSMA3-AS1 | 0.416991 | 6.94E-45 | postive |
| METTL14 | PSMA3-AS1 | 0.451185 | 3.83E-53 | postive |
| METTL3 | PSMA3-AS1 | 0.50243 | 2.02E-67 | postive |
| RBM15 | PSMA3-AS1 | 0.536023 | 3.44E-78 | postive |
| YTHDC1 | PSMA3-AS1 | 0.447134 | 4.09E-52 | postive |
| YTHDC2 | PSMA3-AS1 | 0.65916 | 2.90E-130 | postive |
| RBM15 | RAB30-DT | 0.488029 | 3.68E-63 | postive |
| FMR1 | RAB33B-AS1 | 0.51591 | 1.34E-71 | postive |
| METTL14 | RAB33B-AS1 | 0.487016 | 7.20E-63 | postive |
| RBM15 | RAB33B-AS1 | 0.708107 | 1.33E-158 | postive |
| YTHDC1 | RAB33B-AS1 | 0.414879 | 2.10E-44 | postive |
| YTHDC2 | RAB33B-AS1 | 0.615186 | 5.33E-109 | postive |
| FMR1 | RAP2C-AS1 | 0.492781 | 1.52E-64 | postive |
| METTL14 | RAP2C-AS1 | 0.466591 | 3.49E-57 | postive |
| RBM15 | RAP2C-AS1 | 0.515467 | 1.86E-71 | postive |
| YTHDC2 | RAP2C-AS1 | 0.567476 | 1.96E-89 | postive |
| FMR1 | SEPSECS-AS1 | 0.428104 | 1.82E-47 | postive |
| RBM15 | SEPSECS-AS1 | 0.444815 | 1.56E-51 | postive |
| YTHDC2 | SEPSECS-AS1 | 0.490546 | 6.85E-64 | postive |
| METTL3 | SH3BP5-AS1 | 0.41145 | 1.24E-43 | postive |
| RBM15 | SH3BP5-AS1 | 0.402707 | 1.05E-41 | postive |
| YTHDC2 | SH3BP5-AS1 | 0.603339 | 7.87E-104 | postive |
| METTL3 | SNHG12 | 0.488835 | 2.15E-63 | postive |
| HNRNPC | SNHG30 | 0.430996 | 3.74E-48 | postive |
| YTHDC2 | TSPOAP1-AS1 | 0.463182 | 2.85E-56 | postive |
| FMR1 | ZKSCAN2-DT | 0.431609 | 2.67E-48 | postive |
| METTL3 | ZKSCAN2-DT | 0.452427 | 1.84E-53 | postive |
| RBM15 | ZKSCAN2-DT | 0.519044 | 1.35E-72 | postive |
| YTHDC1 | ZKSCAN2-DT | 0.448409 | 1.95E-52 | postive |
| YTHDC2 | ZKSCAN2-DT | 0.570129 | 1.93E-90 | postive |
