# Supplementary material for: Construction and validation of a nomogram based on N6‐Methylandenosine‐related lncRNAs for predicting the prognosis of non‐small cell lung cancer patients
Source: Cancer Med. 2022 Jun 21;12(2):2058–74. doi: 10.1002/cam4.4961 (PMC9883402; doi:10.1002/cam4.4961)
Supplement: Supplementary file 10 — Table S4 [file CAM4-12-2058-s001.docx]

**Table S4. Twelve m^6^ARLncRNAs identified by multivariate Cox proportional hazard regression analysis**

| **lncRNAs** | **coef** | **HR** | **95% CI lower** | **95% CI higher** | | ***P-*value** |
| --- | --- | --- | --- | --- | --- | --- |
| SNHG12 | -0.056 | 0.945 | 0.896 | | 0.997 | 0.037 |
| AP001347.1 | 0.577 | 1.781 | 1.063 | | 2.983 | 0.028 |
| ITGA9-AS1 | -0.895 | 0.409 | 0.146 | | 1.142 | 0.088 |
| AL034550.1 | 0.455 | 1.577 | 1.052 | | 2.364 | 0.028 |
| AC083843.2 | -0.240 | 0.787 | 0.646 | | 0.958 | 0.017 |
| TSPOAP1-AS1 | -0.832 | 0.435 | 0.197 | | 0.959 | 0.039 |
| SNHG30 | -0.069 | 0.934 | 0.900 | | 0.969 | < 0.001 |
| AL021328.1 | -0.466 | 0.628 | 0.428 | | 0.920 | 0.017 |
| AC024060.2 | 0.177 | 1.193 | 1.061 | | 1.342 | 0.003 |
| AL137003.1 | -0.220 | 0.803 | 0.624 | | 1.033 | 0.087 |
| LINC01138 | 0.308 | 1.360 | 1.158 | | 1.598 | < 0.001 |
| SEPSECS-AS1 | -0.947 | 0.388 | 0.191 | | 0.787 | 0.009 |

HR: hazard ratio; CI: confidence intervals.
